# Evolution of Sex-linked Genes and the Role of Pericentromeric Regions in Sex Chromosomes: Insights from Diploid Willows

**DOI:** 10.1093/molbev/msae235

**Published:** 2024-11-12

**Authors:** Yi Wang, Ren-Gang Zhang, Elvira Hörandl, Zhi-Xiang Zhang, Deborah Charlesworth, Li He

**Affiliations:** Eastern China Conservation Centre for Wild Endangered Plant Resources, Shanghai Chenshan Botanical Garden, Shanghai 201602, China; Laboratory of Systematic Evolution and Biogeography of Woody Plants, School of Ecology and Nature Conservation, Beijing Forestry University, Beijing 100091, China; Yunnan Key Laboratory for Integrative Conservation of Plant Species with Extremely Small Populations, Kunming Institute of Botany, Chinese Academy of Sciences, Kunming 650201, Yunnan, China; Department of Systematics, Biodiversity and Evolution of Plants (with Herbarium), University of Göttingen, Göttingen 37073, Germany; Laboratory of Systematic Evolution and Biogeography of Woody Plants, School of Ecology and Nature Conservation, Beijing Forestry University, Beijing 100091, China; Institute of Ecology and Evolution, School of Biological Sciences, University of Edinburgh, Edinburgh EH9 3FL, UK; Eastern China Conservation Centre for Wild Endangered Plant Resources, Shanghai Chenshan Botanical Garden, Shanghai 201602, China

**Keywords:** *Salix*, recombination landscape, inversion, turnover events, transposition

## Abstract

The evolution of sex chromosomes can involve recombination suppression sometimes involving structural changes, such as inversions, allowing subsequent rearrangements, including inversions and gene transpositions. In the two major genus *Salix* clades, *Salix* and *Vetrix*, almost all species are dioecious, and sex-linked regions have evolved on chromosome 7 and 15, with either male or female heterogamety. We used chromosome conformation capture (Hi-C) and PacBio HiFi (high-fidelity) reads to assemble chromosome-level, gap-free X and Y chromosomes from both clades, *S. triandra* (15XY system), a basal species in the *Vetrix* clade, and the *Salix* clade species *S. mesnyi* (7XY system). Combining these with other available genome assemblies, we found inversions within the sex-linked regions, which are likely to be pericentromeric and probably recombined rarely in the ancestral species, before sex-linkage evolved. The Y-linked regions in all 15XY and 7XY species include partial duplicates containing exon 1 of an *ARR17*-like gene similar to male-determining factors in other Salicaceae species. We also found duplicates of a Y-specific gene, which we named *MSF*. The derived *Salix* clade 7XY chromosome systems appear to have evolved when these two genes transposed from the 15Y to the 7Y. Additionally, the 7Y chromosomes in *S. dunnii* and *S. chaenomeloides* probably evolved from the ancestral 7X of the *Salix* clade, involving a similar transposition, and loss of the ancestral 7Y. We suggest that pericentromeric regions that recombine infrequently may facilitate the evolution of sex linkage.

## Introduction

In flowering plants, the predominant reproductive strategy is hermaphroditism (Darwin 1877). However, some plants have evolved separate sexes, a condition known as dioecy (reviewed in Westergaard 1958; Ming et al. 2011; Charlesworth 2013; Renner 2014). If separate sexes evolve from ancestral hermaphroditism (including monoecy) two mutations are required, with the second one being advantageous in one sex, and disadvantageous in the other sex, termed sexual antagonism (SA). If a two factor polymorphism is established, and the mutations are in different genes, selection favors closer linkage between the two, which then behave as a single co-segregating genetic locus (Charlesworth and Charlesworth 1978). Alternatively, one mutation becomes fixed, resulting in a single polymorphic sex-determining gene, or a turnover occurs, with a single sex-determining factor replacing a previous system. Once such a sex-determining “locus” is established, further SA mutations may subsequently arise and establish polymorphisms in the same genome region, again favoring closer linkage, expanding the locus over time. This process may eventually lead to complete recombination suppression, creating Y- and X-linked regions that thereafter evolve without exchanges, termed sex-linked regions (SLRs) or sex-determining loci.

A new sex-determining factor may subsequently appear on another chromosome, including an autosome, or at a new location on a sex chromosome. Such turnover events may involve de novo appearance of a new sex-determining gene, or duplicative transposition inserting a copy of an ancestral gene into a new location (reviewed by Pan et al. 2021; Charlesworth and Harkess 2024), provided that the duplicate gene escapes the most common fate of pseudogenization, and becomes neo-functionalized or sub-functionalized (Moore and Purugganan 2005). In two *Populus* species, a partial duplication of an autosomal gene, *FERR*, is the candidate maleness factor (Xue et al. 2020). In another example, sex chromosome turnovers in the genus *Actinidia* resulted from the movement of sex-determining genes. In this case, the male-specific regions of all species studied share three Y-linked coding genes: *FrBy*, *SyGI*, and *YFT* (Akagi et al. 2023). Such new loci may in turn trigger establishment of SA polymorphisms, and potentially evolution of a nonrecombining region that might undergo genetic degeneration, with loss of genes from the haplotype carried by the heterogametic sex.

**Fig. 1.**
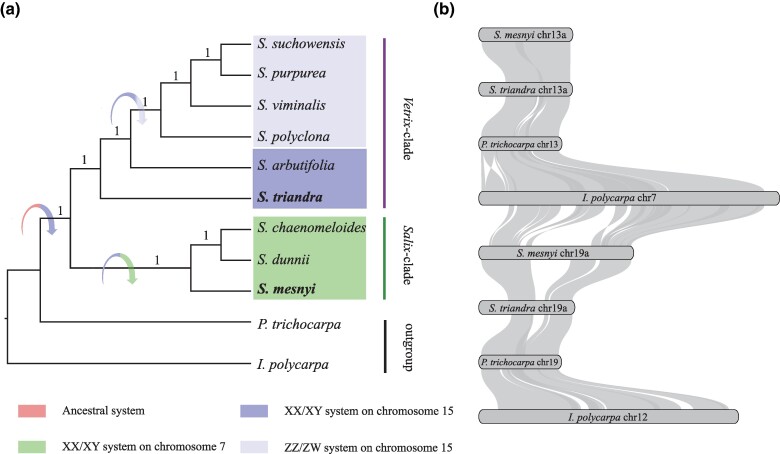
a) Phylogenetic relationships based on single-copy homologous genes from species with assembled genomes in the genus *Salix*, and the most likely turnover events inferred by this study and Xue et al. (2024) indicated by arrows, using *P. trichocarpa* and *I. polycarpa* as outgroups. b) Collinearity analysis between chromosomes 13 and 19 of *S. mesnyi*, *S. triandra*, and *P. trichocarpa*, and chromosome 7 and 12 of *I. polycarpa*.

**Fig. 2.**
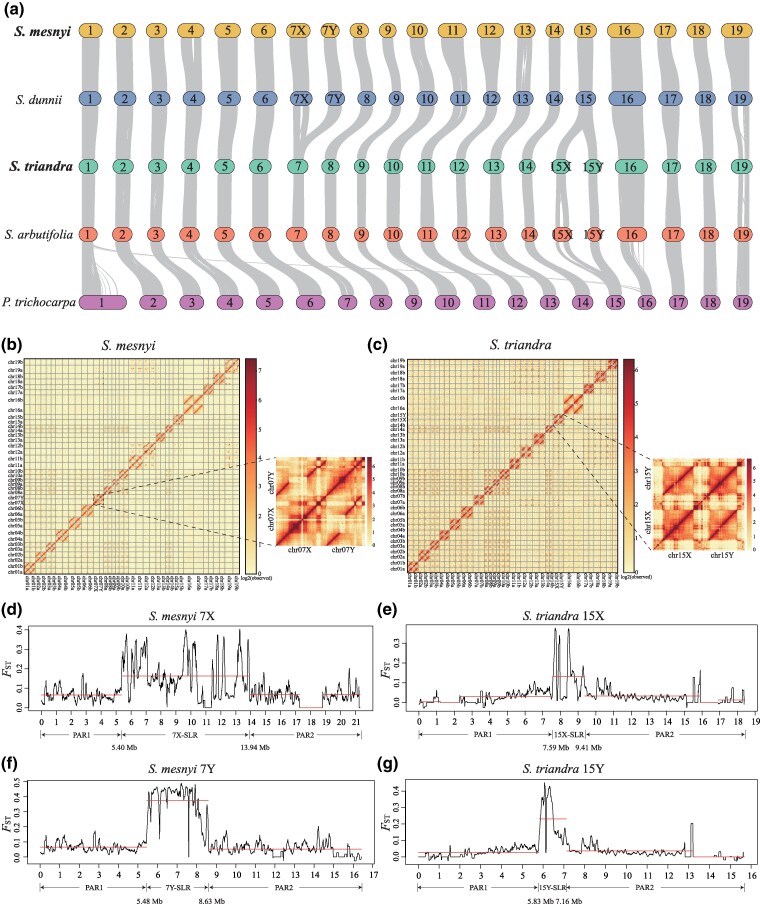
Genomic synteny of *Salix* species and sex chromosomes of *S. mesnyi* and *S. triandra*. a) Comparative synteny analysis of *Salix* genomes. Chromosome IDs are labeled at the middle of each bar. b and c) Genome-wide analysis of chromatin interactions based on Hi-C data for the *S. mesnyi* and *S. triandra* genomes, respectively. d and f) Identification of the *S. mesnyi* 7X and 7Y-SLRs. F_ST_ values between the sexes in 100-kb overlapping windows with 10-kb steps. Red horizontal lines highlight three regions identified by changepoint analysis as having distinct F_ST_ values. e and g) Identification of the *S. triandra* 15X and 15Y-SLRs. The explanations above apply to this plot.

Here, we infer that multiple such changes have occurred in willows (family Salicaceae). Three genera of Salicaceae, *Populus*, *Salix*, and *Idesia* (Figs. 1a and 2a) share a whole genome duplication event, termed the Salicoid WGD (Ma et al. 2013; Gladysh et al. 2024; Ogutcen et al. 2024). Chromosomes 13 and 19 of both *Populus* and *Salix*, are each largely homologous with the chromosomes 7 and 12 in *Idesia polycarpa*, with chromosome 13 of the former species being homologous to chromosome 7 of *I. polycarpa*, and chromosome 19 of the former species being homologous to *I. polycarpa* chromosome 12 (Table 1, Fig. 1a and b) (He et al. 2021; Zuo et al. 2024). In *Populus*, *ARR17*-like partial duplicate sequences in the Y-SLR contain exon 1 and generate small RNAs (sRNAs), via either DNA methylation or mediating cleavage of mRNA, silencing the intact *ARR17*-like gene, which is on chromosome 19; this indirectly triggers the activation of *PISTILLATA* (*PI*), which suppresses female development and is required for stamen development (reviewed in He et al. 2024). Intact *ARR17*-like genes trigger female development when turned on and male development when turned off in the presence of the sex-linked partial duplicates (Muller et al. 2020). The partial duplicate therefore acts as a master sex-determining factor. The intact *ARR17*-like gene is also on chromosome 19 in species of *Salix*, the sister genus to *Populus*, but the fate of the homeologous chromosome 13 copy created by the WGD is unclear in *Salix*, and whether it is involved in sex determination.

**Table 1:** The relationship between the sex-determining candidates in *Salix* species and the two outgroup species

		Chromosome carrying genes of interest				
Genus or species	Clade	Sex determination system	*ARR17*-like gene		*MSF* (this study)	
			Intact (progenitor)	Partial duplicates with exon 1	Intact (progenitor, 14 exons)	Duplicates (5 exons)
*Salix*	*Salix*	7XY	2 copies on chr19	chr07	chr13	chr07
	*Vetrix*	15XY and 15ZW	2 copies on chr19	chr15	chr13	chr15
*Populus trichocarpa*	…	19XY	One copy on chr19 and one on chr13	chr19	chr13, chr19	NA
*Idesia polycarpa*	…	Unknown	One copy on chr12 and one on chr7	chr20	chr7, chr12	NA

Note: Chr13 and chr19 of *Populus* and *Salix*, along with chr7 and chr12 of *Idesia polycarpa* are homologous chromosomes created by the Salicoid WGD, see Fig. 1b. Chromosome 12 of *Idesia polycarpa* is homologous to chromosome 19 of *Populus* and *Salix*, while chromosome 7 of *Idesia polycarpa* is homologous to chromosome 13 of these genera.

The genus *Salix* includes two major clades: *Salix* and *Vetrix* (Fig. 1a). The two species *S. arbutifolia* and *S. triandra*, appearing on basal branches of the *Vetrix* clade, have XY systems on chromosome 15 (Wang et al. 2023, 2024a), while the later-branching species *S. purpurea* and *S. viminalis* have ZW systems, also on chromosome 15 (Almeida et al. 2020; Zhou et al. 2020). The *Salix* clade species *S. dunnii* and *S. chaenomeloides* have a XY system on chromosome 7 (He et al. 2021; Wang et al. 2022). How these changes in the sex-determining region and the heterogamety occurred is largely unknown. The phylogeny of *ARR17*-like partial duplicates in Y-specific regions of chromosomes 15 and 7 of *S. arbutifolia* and *S*. *chaenomeloides* suggested that the 7XY and 15XY systems may share the same sex determination mechanism (Wang et al. 2022), but this needs further confirmation. In addition, it is unclear whether the *Salix* and *Vetrix* clades share other Y-linked sequences, and whether these too moved between the chromosomes of the two clades, in a sex-determining factor turnover, as observed in *Actinidia* (see above).

Here, we describe results from chromosome conformation capture (Hi-C) and PacBio HiFi (high-fidelity) reads to assemble chromosome-level gap-free X and Y chromosome sequences of one species from each clade. *Salix triandra* occupies a basal position in the phylogeny of the *Vetrix* clade, and its sex determination system probably represents the ancestral state of the genus *Salix*, while *S. mesnyi* is basal relative to *S. dunnii* and *S. chaenomeloides* in the *Salix* clade (Wu et al. 2015; Wang et al. 2023). Hence, these two species are well-suited for inferring changes during the evolution of willow sex chromosomes. Combining the new sequences with other willow genome assemblies, from *S. dunnii* (He et al. 2024), *S. chaenomeloides* (Wang et al. 2022), *S. arbutifolia* (Wang et al. 2024a), *S. polyclona* (Wang et al. 2024), *S. purpurea* (Zhou et al. 2020), and two outgroup species within the Salicaceae, *Populus trichocarpa* (https://genome.jgi.doe.gov) and *I. polycarpa* (Zuo et al. 2024), we infer many changes between just the *Salix* species with male heterogamety. The results revealed that:

- Multiple sex chromosome turnover events have occurred in willows. The change from an ancestral system with male heterogamety to one with female heterogamety, has been described elsewhere (Wang et al. 2024a), and we describe two more here.
- A second turnover involved the sex-determining locus changing from the chromosome 15 location, which is ancestral in *Salix*, to a new one on chromosome 7 (Xue et al. 2024). We now show that this change involved transposition as a unit or cassette of two Y-linked factors (the previously known *ARR17*-like partial duplicates containing exon 1, plus a newly discovered sequence that we named *MSF*).
- The ancestral situation within *Salix* reflects a previous insertion of this unit (which is derived from genes on the ancestral sex chromosome 19) into rarely recombining pericentromeric regions, and each event was followed by complete recombination suppression across the region.
- A further turnover is detected in two species, *S. dunnii* and *S. chaenomeloides,* in the *Salix* clade. This involved recent movement of Y-linked male factors to the ancestral 7X, converting it to a neo-7Y.

## Results

### Genome Assembly and Annotation

Our combined sequencing and analyses yielded genome assemblies of male individuals of *S. mesnyi* and *S. triandra*. As described in the Materials and Methods, a haplotype-resolved assembly of the *S. mesnyi* genome was generated by integrating a total of ∼43 Gb of Illumina reads (100× coverage), ∼33 Gb of HiFi reads (80× coverage) with an average length of 17 kb, along with ∼44 Gb of Hi-C reads (supplementary table S1, Supplementary Material online). For the *S. triandra* genome, our assembly is based on ∼48 Gb of Illumina reads (143× coverage), ∼44 Gb of HiFi reads (131× coverage) with an average length of 17 kb, and ∼42 Gb of Hi-C reads (supplementary table S1, Supplementary Material online). The HiFi and Hi-C reads for *S. mesnyi* yielded chromosome-scale assemblies; among the 43 scaffolds, 38 were assigned to the 19 phased chromosome pairs (including gap-free 7X and 7Y, inferred as described below, see Fig. 2a and b), four to the mitochondrial (Mt) genome, and one to the chloroplast (Cp) genome (supplementary tables S2 and S3, Supplementary Material online). For *S. triandra*, a total of 40 scaffolds were obtained, 38 corresponding to the 19 chromosomes (phased and gap-free for 15X and 15Y, see Fig. 2a and c), plus the Mt and Cp genomes (supplementary tables S2 and S3, Supplementary Material online). Synteny and collinearity analyses detected no inter-chromosomal rearrangements between the two clades; all chromosomes show one-to-one homologous relationships (Fig. 2a). The arrangements of genes within chromosomes are also generally highly conserved (Fig. 2a). Overall, the genome assemblies of the two species are highly accurate, consistent, and contiguous, and those of the sex chromosomes are gap-free, as are the autosomes, with few exceptions (Fig. 2a to c, supplementary table S4, Supplementary Material online). The total assembly sizes of *S. mesnyi* and *S. triandra* are 794 Mb and 672 Mb, respectively (supplementary table S2, Supplementary Material online).

Based on transcriptome data, and on protein homology-based and ab initio predictions, we identified 32,621 gene models in the *S. mesnyi* haplotype *a* and 32,431 in haplotype *b*, plus 74 in the Cp and 147 in the Mt genomes (supplementary table S3, Supplementary Material online). In *S. triandra*, we identified 29,454 and 28,805 gene models in haplotypes *a* and *b*, respectively, plus 83 in the Cp and 117 in the Mt genome (supplementary table S3, Supplementary Material online). The genome assemblies of these two species respectively include 98.4% and 98.3% of the complete conserved BUSCO genes in the embryophyta_odb10 database. We estimate that 383.09 Mb of the *S. mesnyi* sequence is repetitive (48.24% of the total genome assembly) and 310.57 Mb in *S. triandra* (46.23%). The predominant types of repetitive sequences in both species are LTRs, accounting for 22.9% and 32.6% of the repetitive sequences in *S. mesnyi* and *S. triandra*, respectively (supplementary table S5, Supplementary Material online).

### Sex-linked Regions of *S. mesnyi* and *S. triandra*

**Fig. 3.**
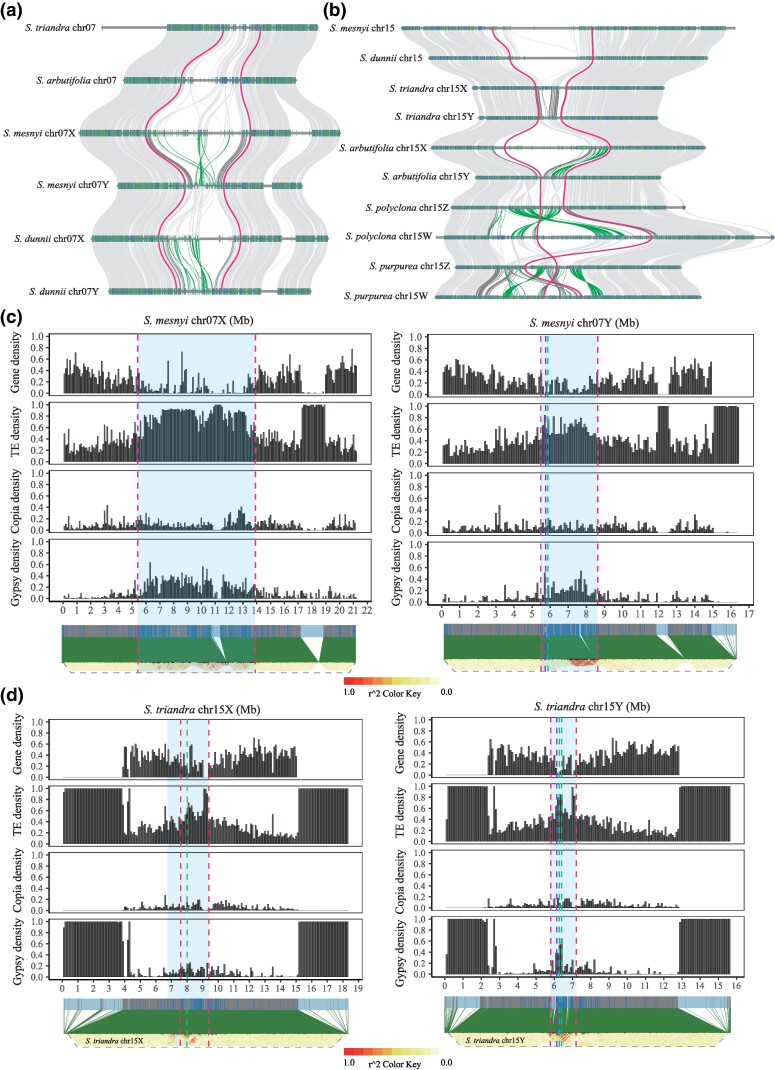
Evolutionary characteristics of sex-linked regions (SLRs) in willows. a and b) Collinearity analyses of chromosomes 7 and 15 of willows. Autosomes are labelled with their numbers, and when a chromosome carries the sex-determining locus, it is labeled as X, Y, Z, or W. The thick lines in (a) indicate the SLRs boundaries in *S. mesnyi* and their homologous regions on the autosomes of other species, while the thick lines in (b) similarly indicate the SLR boundaries in *S. triandra*. The dark grey lines represent collinear genes in the X and Y-SLRs of each species, and dark green indicates inversions in these SLRs. c and d) Inference of the pericentromeric regions of the *S. mesnyi* and *S. triandra* sex chromosomes, indicated as shaded areas. Each panel shows estimated gene densities for the X (left) and Y (right) chromosomes, and the total densities of all TEs, LTR-Gypsy and LTR-copia TEs, and LD values (estimated as *r*^2^ values between all pairs of loci). The outer dotted lines indicate the SLR boundaries. The purple and blue dotted lines in the SLRs of the Y chromosomes of *S. mesnyi* and *S. triandra* show the locations of the *ARR17*-like partial duplicates and *MSF*, respectively. The green dotted line (only in the *S. triandra* 15XY-SLRs), represents the inferred ancestral location of the SCOs mentioned in the text.

Consistent with previous studies of basal species in the *Vetrix* clade (Wang et al. 2023), *S. triandra* also has an XY system on chromosome 15. All species of the *Salix* clade in which sex determining systems have previously been identified (including *S. dunnii* and *S. chaenomeloides*, shown in Fig. 1a, and *S. nigra*), as having XY systems involving chromosome 7 (He et al. 2021; Sanderson et al. 2021; Wang et al. 2022), and our sequences of *S. mesnyi* confirm this for this species too. The evidence for these conclusions in the newly sequenced species is based on using the chromosome quotient (CQ) approach to distinguish the X and Y haplotypes in the phased assemblies. We identified regions with CQ close to 0 in haplotype 7*b* of *S. mesnyi* and haplotype 15*b* of *S. triandra*, indicating that both these species have XY systems (supplementary figs. S1 to S3, Supplementary Material online). Within the region that shows sex linkage (see below), we determined that the haplotypes 7*a* and 15*a* include the X-linked regions, and 7*b* and 15*b* the Y-linked regions (CQ close to 0; supplementary figs. S1 to S3, Supplementary Material online). Across chromosome 7, haplotypes *a* and *b* are largely syntenic in the *Salix* clade species (Fig. 3a). The haplotypes across chromosome 15 are also largely syntenic in the *Vetrix* clade species (Fig. 3b).

To localize these sex-linked regions in the X and Y chromosome assemblies of *S. mesnyi* and *S. triandra*, we used F_ST_ estimates from the short reads (in *S. mesnyi*, 4,575,184 SNPs were found in sequences assigned to haplotype *a*, and 4,555,411 were assigned to haplotype *b*; *S. triandra* yielded 3,468,553 haplotype *a* SNPs, and 3,473,509 *b* ones; see Materials and Methods and supplementary tables S6 to S8, Supplementary Material online). We estimated F_ST_ values based on these four datasets individually, because the X- and Y-linked regions of a species differ in size. In *S. mesnyi*, changepoint analysis identified regions with high F_ST_ values in an 8.54 Mb region between 5.40 and 13.94 Mb of 7*a*, the 7X-SLR (Fig. 2d, supplementary fig. S4, Supplementary Material online), and a 3.15 Mb region of 7*b* (5.48 to 8.63 Mb) of the 7Y-SLR (Fig. 2f, supplementary fig. S5, Supplementary Material online). These SLRs are larger than in the other *Salix* clade species so far studied, *S. dunnii* (Fig. 3a). In *S. triandra*, regions with high F_ST_ values are detected between 7.59 and 9.41 Mb of haplotype 15*a* (1.82 Mb, Fig. 2e, supplementary fig. S6, Supplementary Material online) and 5.83 to 7.16 Mb of 15*b* (1.33 Mb, Fig. 2g, supplementary fig. S7, Supplementary Material online) of the 15X and 15Y-SLRs, respectively. In *S. triandra*, these regions are smaller than the SLRs of the other *Vetrix* clade species (Fig. 3b), *S. arbutifolia* (15XY) and *S. polyclona* (15ZW); we did not include *S. purpurea* in this comparison, because its 15W- and 15Z-SLRs have not yet been assembled from PacBio HiFi sequences (Zhou et al. 2020; Hyden et al. 2023).

In both *S. triandra* and *S. mesnyi*, the SLRs are in the centers of their sex chromosomes, which are metacentric (Levan et al. 1964), and these are probably pericentromeric regions, based on high TE densities and correspondingly low gene densities in both the X and Y haplotypes (Fig. 3a to d, supplementary figs. S8 and S9, Supplementary Material online). Linkage disequilibrium (LD) is high (*r*^2^ often close to 1) across both these SLRs. In contrast, the flanking regions on each side have lower *r*^2^ values, indicating that they recombine, as expected for pseudo-autosomal regions (PARs) (Fig. 3c and d, supplementary fig. S10, Supplementary Material online). In both species, LD decays rapidly to low levels between SNPs in autosomal and PARs, and much more slowly in the X-SLRs, consistent with the conclusion that the X-SLRs are pericentromeric regions that recombine rarely. Decay of LD in the Y-SLRs occurs even more slower than in their X counterpart regions, indicating that the observed LD does not solely reflect their pericentromeric locations; SNPs separated by more than about 25 kb maintain high *r*^2^ values without consistent decay, in strong contrast with the X-SLRs, indicating that Y-SLRs recombine less often (supplementary fig. S10, Supplementary Material online). The *S. triandra* 15X- and Y-SLRs appear to include most, but not all, of the pericentromeric regions, whereas those of the *Vetrix* clade species previously studied, *S. arbutifolia*, occupy the entire pericentromeric region (Wang et al. 2024a) (Fig. 3d).

### Evolution of Sex-linked Regions in the *Salix* and *Vetrix* Clades

Despite the overall largely syntenic arrangements of the two haplotypes of chromosomes 7 or 15 in our sequenced species, more detailed collinearity analysis showed that, except in *S. triandra*, the SLRs are rearranged due to inversions between the X- and Y-, or Z- and W-linked regions, as well as between these regions and their homologous autosomes, which probably represent their respective ancestral states (Fig. 3a and b, supplementary fig. S11, Supplementary Material online) (Gulyaev et al. 2022; Wang et al. 2024a; Xue et al. 2024). All the inversions are species-specific, and none of them is shared between species. Importantly, rearrangements are significantly associated with sex linkage: between the three *Vetrix* clade species, *S. triandra*, *S. arbutifolia*, and *S. polyclona*, in which chromosome 7 is an autosome, only one inversion was found in the inferred pericentromeric region of *S. polyclona* haplotype *a* and *b*, and none were detected on other autosomes (Fig. 3a, supplementary figs. S12a and S13, Supplementary Material online), and similarly for chromosome 15, which is an autosome in the *Salix* clade species, no inversions were detected (Fig. 3b, Table 2, supplementary fig. S12b and table S9, Supplementary Material online).

**Table 2:** Test of the significance of the differences in the number of inversions between sex chromosomes and nonsex chromosomes in the two *Salix* clades

Clade and chromosome	Number of comparisons (see supplementary table S9, Supplementary Material online)	
	Autosomes among different species	Autosomes and sex chromosomes among different species, and sex chromosomes among different species
*Salix*: chr7, *P* = 0.0002***		
Inversions	8	29
No inversions	7	0
*Vetrix*: chr15, *P* = 0.001***		
Inversions	0	25
No inversions	6	9

Note: Asterisks (*) indicate significant differences in the numbers of inversions by Fisher's exact test, with the null hypothesis that SLRs and other genome regions are liable to be inverted to similar degrees. Species and chromosomes tested: *S. triandra* 7*a* and 7*b*, *S. arbutifolia* 7*a* and 7*b*, *S. polyclona* 7*a* and 7*b*, *S. mesnyi* 7X and 7Y, *S. dunnii* 7X and 7Y, *S. mesnyi* 15*a* and 15*b*, *S. dunnii* 15*a* and 15*b*, *S. triandra* 15X and 15Y, *S. arbutifolia* 15X and 15Y, *S. polyclona* 15Z and 15W.

The small size of the *S. triandra* 15X and 15Y-SLRs, estimated as proportions of the entire assembled sex chromosomes, may reflect either a lack of accumulation of repeat sequences in its pericentromeric region, or deletion of repeat-rich regions (supplementary fig. S14 and table S10, Supplementary Material online). In *S. arbutifolia* and the distantly related *S. polyclona*, the proportions of the chromosome 15 assemblies for the 15X-SLR and 15W-SLR are larger than for the *S. triandra* 15X-SLR, and the 15Y-SLR and 15Z-SLR sizes are also larger than the *S. triandra* 15Y-SLR (supplementary table S10, Supplementary Material online). The SLR size differences probably reflect repetitive sequence differences within the SLRs (either due to gains, or to losses after duplicative transposition to other genome regions) and changes in the boundaries causing expansion into either or both flanking regions.

In the 15XY species *S. arbutifolia*, heteromorphism of X and Y haplotypes was attributed to X expansion involving evolution of large arrays of repetitive sequences (termed an “X-extended region”), and other gene duplication events, rather than Y degeneration (Wang et al. 2024a). The X-SLRs are also larger than the Y-SLRs in the other 15XY species, *S. triandra*, and also in the 7XY species *S. mesnyi* and *S. dunnii* (supplementary fig. S14, Supplementary Material online). The main elements within the expanded X-SLR region are again repetitive sequences and duplicated genes (supplementary fig. S14, Supplementary Material online). This unexpected expansion of the X-SLRs is discussed below.

### Neo-Y Evolution in the 7XY Clade

**Fig. 4.**
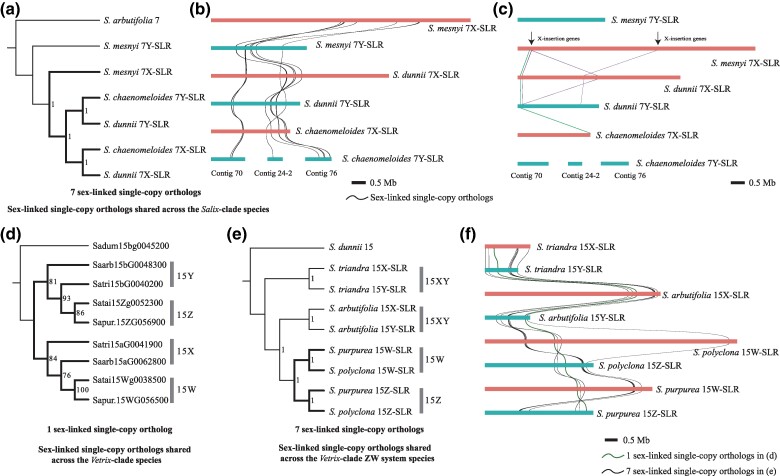
Different evolutionary patterns of *Salix* sex chromosomes. a) The ASTRAL species tree based on seven sex-linked single-copy orthologs (SCOs). b) Collinearity analysis of 7X- and 7Y-SLRs based on these seven SCOs. The size of the bars indicates the SLR lengths. c) Collinearity of X specific genes inserted into the 7X-SLR of *S. mesnyi* and the genes within SLRs of *S. dunnii* and *S. chaenomeloides*. The leftmost line represent collinearity with *S. dunnii* 7X-and 7Y-SLRs and *S. chaenomeloides* 7X-SLRs, and other lines represent collinearity only with *S. dunnii* 7X- and 7Y-SLRs. d) Gene tree of one sex-linked SCO consistent with recombination suppression before the divergence of *Vetrix* clade species. e) The ASTRAL species tree based on seven sex-linked SCOs in the SLRs of the *Vetrix* clade, suggesting that recombination may have stopped after the two ZW species *S. polyclona* and *S. purpurea* split from the XY species *S. triandra* and *S. arbutifolia* (whose orthologs did not become fully sex-linked). f) Collinearity analysis of 15X-, 15Y-, 15W-, and 15Z-SLRs based on eight SCOs of *Vetrix* clade species. The green line represents the single-copy ortholog in (d), and the black line represents the seven SCOs in (e).

As all three *Salix* clade species, *S. mesnyi*, *S. dunnii*, and *S. chaenomeloides*, have 7XY systems, they might all share a common ancestral 7X- and 7Y-SLRs. To identify single-copy orthologs (SCOs) that are in the SLRs, but not candidate sex-determining genes, for phylogenetic analyses, we used the haplotype *a* assembly of chromosome 7 of the *Vetrix* clade species *S. arbutifolia*, which is an autosome in this species, as an outgroup. We ascertained a total of 20 such genes that are within all three fully sex-linked regions. These genes appear in two distinct regions within each SLR, separated by a gap (as described above), and their orientations are affected by the inversions already mentioned within the SLRs (Fig. 4a and b). Since the 7Y-SLR of *S. chaenomeloides* is not fully assembled, and consists of three 7Y-specific contigs (Contig24-2: 500,000 bp; Contig70: 1,113,471 bp; Contig76: 860,332 bp) (Wang et al. 2022), the size of the region shared with the *S. dunnii* 7Y is not certain. Unexpectedly, none of the genes yielded the tree expected if the 7XY-SLRs share a common ancestor (the 7X gametologs of the three ingroup species should form one cluster and their 7Y sequences a separate cluster). Instead, the most frequent topology (seven SCOs) clustered the 7X sequences and 7Y sequences of *S. dunnii* and *S. chaenomeloides* together, sister to the *S. mesnyi* 7X, with the *S. mesnyi* 7Y sequences as an outgroup (Fig. 4a, supplementary table S11, Supplementary Material online). This supports the hypothesis that the *S. dunnii* and *S. chaenomeloides* 7X and 7Y both evolved from a state like that of the *S. mesnyi* 7X, rather than their Ys having evolved from ancestral 7Y chromosome. The other 13 SCOs (whose topology differs) could have recombined between an ancestral 7X and 7Y. The separation of the *S. dunnii* and *S. chaenomeloides* 7Xs from their Ys (Fig. 4a) also suggests that the region stopped recombining in these species since this event in their ancestor. To test this turnover hypothesis further, we identified 7X- and 7Y-SLR specific genes in *S. mesnyi*. We found four genes present in the *S. mesnyi* 7X-SLR but not in the 7Y counterpart or the 7*a* of *S. arbutifolia*, and therefore presumably inserted into the X sequence. Additionally, three of these four 7X-specific insertions have homologous sequences in both the 7X- and 7Y-SLRs of *S. dunnii*, while one gene has no copy in the *S. chaenomeloides* 7Y-SLR, but has homologs in both the *S. dunnii* 7XY-SLRs and *S. chaenomeloides* 7X-SLR (Fig. 4c, supplementary table S12, Supplementary Material online). In the *S. dunnii* 7XY-SLRs, these four genes are physically close to the seven SCOs that suggest an ancestral 7X-SLR, and the two Y-linked candidate male-determining factors described below are also nearby (supplementary table S13, Supplementary Material online). We conclude that the 7X and 7Y of *S. dunnii* and *S. chaenomeloides* probably arose from a 7X similar to that in *S. mesnyi*, in a single event that transferred Y-linked male-determining factors to the 7X. Using the same outgroup, we detected gene losses from both the 7X- and 7Y-SLRs in *S. dunnii* (1.22% and 1.22%, respectively) and more prominently in *S. mesnyi* (5.76% and 4.62%, respectively), consistent with a more recent origin of the *S. dunnii* 7Y-linked region (supplementary fig. S15 and table S14, Supplementary Material online).

### Ancestral Sex-linked Regions Have Been Maintained in the 15XY and ZW Species

In the *Vetrix* clade, Wang et al. (2024a) found that *S. arbutifolia* and *S. purpurea* share two sex-linked genes, despite having 15XY and 15ZW systems, respectively. With our new sequences, we can now make a tree based on these SLR sequences from four species of this clade. The orthologs of only one of these genes (the *S. triandra* sequence Satri15aG0041900 and its orthologs) now cluster by gametologs (Fig. 4d); for this gene, the 15Ys of *S. triandra* and *S. arbutifolia* group with the 15Zs of *S. purpurea* and *S. polyclona*, while their 15Xs group with the *S. purpurea* and *S. polyclona* 15Ws (Fig. 4d), further supporting 15X → 15W and 15Y → 15Z transitions in this clade. The ancestor of the Satri15aG0041900 gene must have been present in a small ancestral *Vetrix* clade SLR, which must have stopped recombining before the basal species *S. triandra* diverged from other *Vetrix* clade species (Fig. 1a). Seven other SLR genes yielded trees in which their 15W and 15Z gametolog pair sequences formed subclades that suggest recombination cessation before the divergence of *S. polyclona* and *S. purpurea*, while the 15X and 15Y sex-linked sequences of *S. triandra* and *S. arbutifolia* cluster by species (Fig. 4e, supplementary table S15, Supplementary Material online). The *S. triandra* sequence Satri15aG0041900 and its orthologs are located in the gap in the SLR assemblies between these seven SCOs and the single SCO indicated in Fig. 4f. Recombination cessation may therefore have expanded to both sides of this gene.

### Candidate Mobile Sex Factors in Willows

**Fig. 5.**
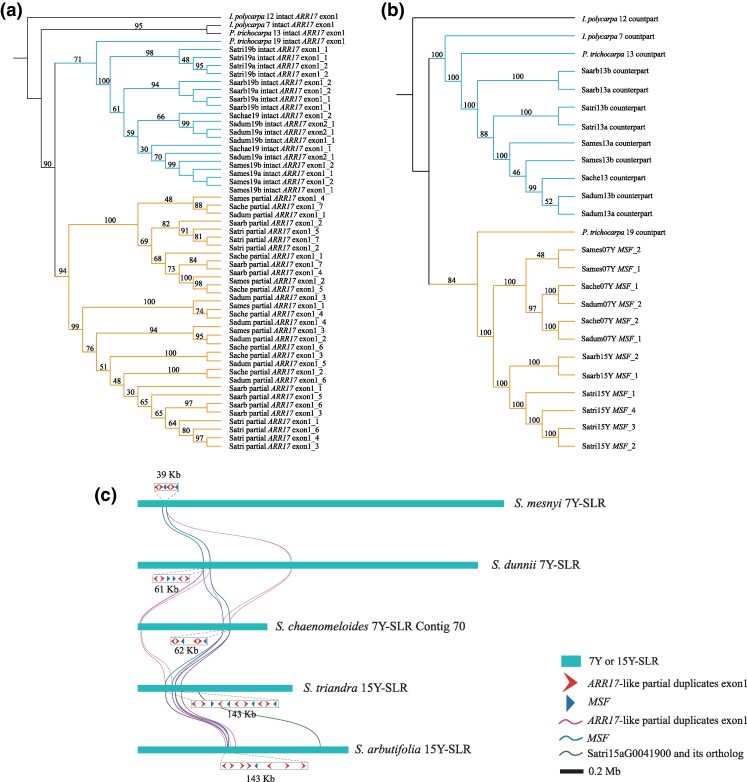
Evolution of *ARR17*-like duplicates and *MSF* genes in *Salix*. a) Phylogenetic tree of exon 1 of the *ARR17*-like gene in two clades of *Salix*. The tree was rooted using the intact exon 1 of the *ARR17*-like gene from chromosome 12 of *I. polycarpa*. b) Phylogenetic relationships of the *MSF* sequences and their counterparts on chromosome 13 and 19 identified in *Salix* species and two outgroup species. All *MSF* and counterparts include exons 1, 2, 3, 5, and 6. Species abbreviations apply to (a and b): Sames: *S. mesnyi*; Sadum: male of *S. dunnii*; Sache: *S. chaenomeloides*; Satri: *S. triandra*; Saarb: *S. arbutifolia*; c) The physical location and collinearity of the exon 1 of *ARR17*-like partial duplicates and *MSF* on chromosomes in *Salix*. The length of the bar represents the length of SLRs.

Intact *ARR17*-like genes can be identified on chromosome 19 in all 7XY and 15XY willows so far studied (whereas *Populus* has a single one on chromosome 19, and its homeolog, chromosome 13, also carries a single intact copy, see Table 1). Like *Populus*, willows also have sex-linked *ARR17*-like partial duplicates, which are in either the 7Y- or the 15Y-SLRs (supplementary tables S16 and S17, Supplementary Material online). In *Populus* species, partial duplicates that include exon 1 are believed to play a role in male determination (Muller et al. 2020; Wang et al. 2022). In a phylogenetic tree using 82 to 107 bp sequences of exon 1, the partial *ARR17*-like duplicates in the *Salix* 7Y- and 15Y-SLRs form a distinct clade (yellow lines in Fig. 5a), supporting a single ancestral origin, consistent with previous findings (Wang et al. 2022). The intact *ARR17*-like gene exon 1 sequences on chromosome 19 of *Salix* and *P. trichocarpa* formed a separate clade (blue lines in Fig. 5a). These results suggest that the exon 1 of *ARR17*-like partial duplicates on Y chromosomes of *Salix* originated from an ancestral chromosome 19 sequence.

In addition to the Y-linked *ARR17*-like partial duplicates, some of which are probably involved in sex determination, as noted above, we detected a gene that is male-specific in *Salix* as it is shared by the Y-SLRs of 7XY and 15XY species. We named this *MSF* (male-specific factor). *MSF* is located close to the *ARR17*-like partial duplicates in the Y-SLRs of these *Salix* species (Fig. 5c, supplementary table S15, Supplementary Material online). In the *Vetrix* clade species *S. triandra*, *MSF* is close to its Satri15aG0041900 gene described above. In the 15Y-SLR of *S. arbutifolia*, in the same clade, *MSF* and the orthologous gene (Saarb15bG0048300) are not physically close, due to an inversion (Wang et al. 2024a) (Fig. 5c, supplementary table S15, Supplementary Material online). *MSF* has a single paralog on chromosome 13 of all *Salix* species so far studied, which has 14 exons in our annotation (supplementary tables S18 and S19, Supplementary Material online). Homologs with 14 exons were also detected on the homologous chromosomes 13 and 19 in the outgroup species, *P. trichocarpa*, as well as the homologous *I. polycarpa* chromosomes 7 and 12 (Table 1, supplementary table S20, Supplementary Material online). A phylogenetic tree of the *MSF* sequences includes the *Salix* species’ 7Y and 15Y *MSFs* and their chromosome 19 *P. trichocarpa* counterparts in a single clade (Fig. 5b, yellow lines), sister to the *Salix* chromosome 13 copies and their counterparts in the two outgroup species. However, the Y-SLR *MSF* sequences include only five exons, and are therefore partial duplicates, similar to the situation for the *ARR17*-like sequences.

**Fig. 6.**
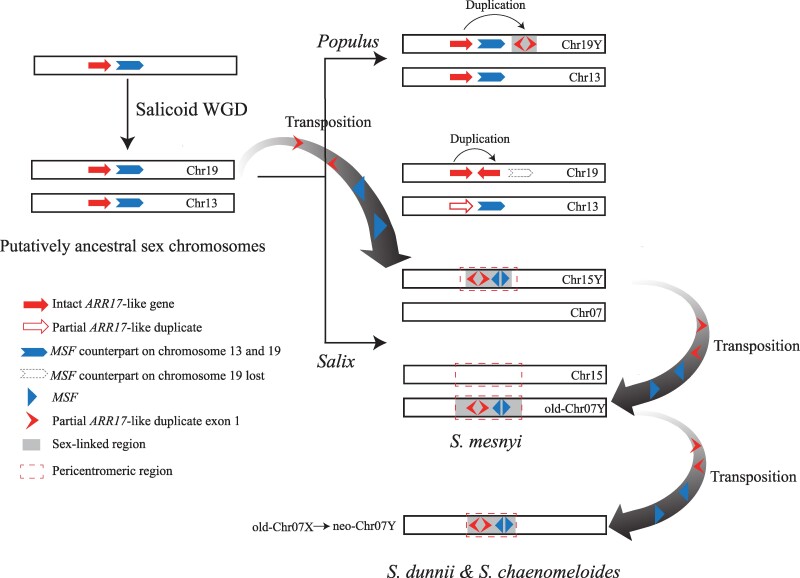
Model for the evolutionary path of sex chromosomes in male heterogametic diploid *Salix*. The black rectangles represent chromosomes. The length of black rectangles is represented as virtual rather than actual length. The gray regions on the chromosomes represent the sex-linked regions, and the dashed boxes indicate the inferred pericentromeric regions. Their lengths are all virtual. All chromosome numbers are labeled on the right side. Solid arrows indicate intact *ARR17*-like genes, while hollow arrows represent partial *ARR17*-like duplicates missing any of the five exons from the intact *ARR17*-like genes. Inverted V-shape arrows represent exon 1 of the partial *ARR17*-like duplicates alone. Solid chevron with a rectangular base arrows represent the *MSF* counterpart with 14 exons on chromosomes 13 and 19, while hollow dashed chevron with a rectangular base arrows indicate that the *MSF* counterpart has been lost. Solid triangles represent the *MSF* genes. For the specific copy numbers of partial *ARR17*-like and *MSF* sequences of studied willows sex chromosomes, see Fig. 5c.

The *MSF* duplicates could potentially be involved in sex determination in willows. The *MSF* homolog in *Arabidopsis thaliana* encodes a DEAD-box ATP-dependent RNA helicase. Deletion of *AIP1*/*AIP2* (*DEAD-box ATP-dependent RNA helicases*) in rice leads to tapetum degradation, impairing pollen development and resulting in male sterility (Li et al. 2011) in a manner similar to that of the kiwifruit male determining factor, *FrBy* (Akagi et al. 2019). The *MSF* gene may thus be important for male function in willows. A higher proportion of ancestral genes has been lost from the X and Y-SLRs in the 15XY species than from those with 7XY systems (supplementary fig. S15 and table S14, Supplementary Material online), further supporting the transition of 15XY → 7XY (Xue et al. 2024). These results suggest the surprising conclusion that the *ARR17*-like partial duplicates carrying exon 1 and *MSF* partial duplicates are both parts of a mobile male-specific unit that was duplicated and translocated from chromosome 19 to 15 in a *Salix* ancestor, and moved from 15 to 7 in an ancestor of the *Salix* clade species, and from 7Y to 7X in an ancestor of *S. dunnii* and *S. chaenomeloides* (Figs. 5c and 6).

### Expression Patterns of *ARR17*, *MSF*, and *PI*-like Duplicates in *S. triandra* and *S. dunnii* Buds and Catkins

To test whether the genes just described are indeed likely to be involved in sex determination, we studied expression in *S. triandra* flower buds. The two intact chromosome 19 *ARR17*-like genes (especially exon 1) are expressed specifically in female buds, but not in male buds (supplementary fig. S16a and b, Supplementary Material online). We also detected high expression of small RNAs (sRNAs) sequences from DNA near the *ARR17*-like partial duplicates within the *S. triandra* 15Y-SLR that include exon 1 (supplementary fig. S16c, Supplementary Material online), suggesting that these may suppress the expression of intact *ARR17*-like genes in males, as it occurs in *Populus* species (Muller et al. 2020; Wang et al. 2022). As the partial *MSF* gene copies are Y-linked in *S. triandra*, they are expressed only in males. In this species, we detected high *MSF* expression in male flower buds and less in male catkins at the time when their intact *ARR17*-like gene expression ceases (supplementary fig. S16d and table S21, Supplementary Material online), consistent with sex determination being complete before catkins develop. The *MSF* and intact *ARR17*-like genes thus appear to have the same temporal expression patterns in males and females. Another gene that shows high expression in *S. triandra* males is a *PI*-like gene that is important in stamen development in *P. tremula* (Leite Montalvao et al. 2022), *P. balsamifera* (Cronk et al. 2020), and *Diospyros lotus* (Yang et al. 2019). Its expression is barely detectable in any of the stages studied in females (supplementary fig. S16e and table S22, Supplementary Material online). The expression patterns of these three genes are similar in *S. dunnii* (supplementary table S23, Supplementary Material online) (He et al. 2024; Wang et al. 2024b). We conclude that exon 1 of *ARR17*-like partial duplicates act as male-determining factors in *Salix*, while *MSF* and *PI*-like genes, with male-specific expression, may be involved in male fertility functions. Further studies are needed to test this hypothesis.

## Discussion

### Turnovers in Willows and a Mobile Sex Determining Unit

The previous section described evidence for several independent changes affecting the sex chromosomes of different groups of *Salix* species. They all appear to involve changes in the pericentromeric regions of chromosomes, consistent with such regions being prone to undergo rearrangements. The gene movements revealed by our sequences might be promoted by actions of transposable elements, or permitted due to low gene densities in such regions. We indeed detect high repetitive sequence densities in the regions identified as SLRs, but our synteny analyses provide no evidence for genetic degeneration in terms of gene losses (Fig. 3, but see supplementary table S14, Supplementary Material online), though the pericentromeric regions showing complete sex linkage have low gene densities and high repeat densities, making such analyses difficult. Figure 6 summarizes the changes detected. The intact *ARR17*-like factor is probably essential for female functions. It remained on chromosome 19, while the homologous chromosome 13 has lost any functional copy in willows. The expression of the intact *ARR17*-like gene is probably suppressed by expression of the *ARR17*-like gene exon 1 present in the partial duplicates in the Y-SLRs on chromosome 7 of *Salix* clade species, and chromosome 15 of *Vetrix* clade 15XY species. The specific expression of intact *ARR17*-like genes in the female buds of *S. dunnii* (supplementary table S23, Supplementary Material online) (He et al. 2024; Wang et al. 2024b), *S. chaenomeloides* (Wang et al. 2022), *S. triandra*, and *S. arbutifolia* (Wang et al. 2022), along with the sRNA produced from exon 1 sequences of *ARR17*-like partial duplicates within Y-SLRs, is consistent with previous evidence that *ARR17*-like genes are involved in sex determination in the Salicaceae (Muller et al. 2020). *MSF* genes may also contribute to sex determination, or may act down-stream. Expression of the single chromosome 13 paralog of *MSF* does not differ between the sexes (supplementary table S21, Supplementary Material online). The sex-linked *MSF* duplicates may have gained a new function in male development, by neo-functionalization or sub-functionalization. Further studies are needed to test the functions of the *MSF* copies.

Inference of the system ancestral to the 7XY and 15XY species is, however, complicated, because these are in two clades. Several observations described above suggest that 7XY systems may be younger than 15XY ones (Xue et al. 2024), but this is not certain, and further investigation of basal species within the *Salix* clade is needed to test this further. The sex determining unit also included the newly discovered *Salix MSF* sequences, as these are found in all Y-SLRs studied (Fig. 5c). Our phylogenetic analysis indicates that both Y-linked partially duplicated genes originated from the ancestral chromosome 19 copies (the *MSF* counterpart has been lost on chromosome 19 of *Salix*). These changes probably created the ancestral 15XY system, which persisted in the *Vetrix* clade, while in the *Salix* clade, the *ARR17*-like partial duplicates containing exon 1, and also *MSF*, moved to chromosome 7 by duplicative transposition, creating the 7XY system (Fig. 6). The close physical proximity of the *ARR17*-like and *MSF* duplicates in the Y-SLRs suggests that these two elements translocated as a single unit between chromosomes 15 and 7, similar to the translocations detected in kiwifruit (Akagi et al. 2019). Within the *Vetrix* clade (Fig. 1a), a phylogenetic analysis inferred another change, a transition from a 15XY to a 15ZW system (Wang et al. 2024a), and our new results support this (Fig. 4d). The *ARR17*-like partial duplicates and *MSF* sequences in the 15Y-SLRs were then inherited by the 15Z of the *Vetrix* clade 15ZW species (supplementary table S18, Supplementary Material online) (Wang et al. 2023; He et al. 2024).

Movements of male-determining factors are well documented in the housefly (Meisel et al. 2017, 2020), resulting in the presence of the same factor on different chromosomes in different populations, rather than a new maleness factor evolving (Meisel et al. 2020). Similar movements of sex-determining factors are documented in the plant genus *Fragaria* (Goldberg et al. 2010; Cauret et al. 2022), involving mobile regions of several kilobases. In *Salix*, also, it appears that no new maleness factor has evolved; instead, the original male-determining *ARR17*-like partial duplicate has moved. The *MSF* gene has moved along with the *ARR17*-like sequences, and duplicated copies are also found on the *Salix* sex chromosomes 15 and 7. The size of the apparently mobile region is not yet clear, as insertions into the pericentromeric regions appear to have been followed by amplification of copy numbers, and rearrangements, probably because the insertions prevented recombination in the Y-linked regions.

In the *Salix* clade, our results also suggest yet another type of turnover, in which an ancestral X chromosome in an ancestral species gave rise to the X and Y chromosomes found in two extant species, perhaps by a similar kind of movement, though not involving a different chromosome. Both the source location and that of the new factor are within the chromosome 7 pericentromeric region, though the copy numbers differ. This proposal resembles the hypothesis suggested for the guppy (Charlesworth et al. 2021), involving the X acquiring a male-determining factor or factors. A similar change is plausible in the plant genus *Spinacia* (She et al. 2023). Our phylogenetic analyses of the *ARR17*-like partial duplicates containing exon 1 and the *MSF* sequences (Fig. 5a and b) in the *Salix* clade suggest that *S. dunnii* and *S. chaenomeloides* acquired both these likely masculinizing factors before their divergence, rather than independently from the autosome 19 where source copies are found. The male-determining factor was probably then transferred (by an as yet unknown mechanism) from the ancestral Y chromosome to the ancestral X chromosome before the ancestral Y was lost in an ancestor of *S. dunnii* and *S. chaenomeloides*.

### The Sex-linked Regions in Willows are Pericentromeric and May Have Evolved Suppressed Recombination

Recently evolved SLRs are predicted to be genetically small regions, for example, single genes involved in turnovers creating new male- or female-determiners (Pan et al. 2021). However, a genetically small region need not be physically small, and once the sex-determining “locus” is established, further SA mutations may subsequently arise that may favor further linkage. These changes may evolve within physically large rarely recombining regions. The *Salix* genus is an example where both the sex-determining gene *ARR17*-like partial duplicates and the male-specific gene *MSF* are found in a region that is pericentromeric in the ancestral autosome (Figs. 3c, d and 5c; supplementary fig. S13, Supplementary Material online) (Wang et al. 2024a). The physically small Y-linked region in cultivated spinach is also within a pericentromeric region (Ma et al. 2022; She et al. 2023). Our Y–X divergence results, and the observation that rearrangements are significantly associated with sex chromosomes after a sex-determining function arises on chromosomes (Table 2), suggest that recombination suppression has evolved in both the X- and Y-linked regions, and Z and W ones, in the species studied. *MSF* could be a sexually antagonistic factor involved in promoting some male function, and perhaps favoring recombination suppression. These events affected the pericentromeric regions of the respective chromosomes. Such regions recombine rarely, and the ancestral species would already have evolved high repeat densities and low gene densities (supplementary fig. S13, Supplementary Material online). The acquisition of a sex-determining factor together with the *MSF*, with male-specific expression, would have created selection for even lower recombination rates. In both the *Vetrix* and *Salix* clades, these changes were followed by rearrangements in the pericentromeric SLRs, but not their homologous autosomal regions, although these are also repeat-rich, which will make rearrangements more likely than in other genome regions. However, as the rearrangements are species-specific, they may be recent, and have occurred after recombination was suppressed. Based on the rearranged regions, recombination suppression appears not to have extended into regions outside the pericentromeric regions.

All the willow X-SLRs are larger than their Y-linked counterparts. Not surprisingly, given their pericentromeric locations (supported by their repeat-richness, see Fig. 3c and d), they also include some inversions, although fewer than in the Y-SLRs (supplementary fig. S11, Supplementary Material online). The size difference is maintained in the 15ZW species that we infer evolved from 15XY systems: in both ZW species, the X-derived W-SLR is larger than the Z one. The previous conclusion for *S. arbutifolia* (Wang et al. 2024a) is thus not an isolated unusual case. The smaller Y-linked regions in *Salix* XY species conform to the generally observed pattern that fully Y-linked regions are often degenerated or partially degenerated. Degeneration and loss of Y-linked genes will allow some regions to be deleted (reviewed by Bergero and Charlesworth 2009). In *Salix*, however, expanded X-linked regions also appear to be important, and further work is needed to understand these changes. Our studies of sex chromosome turnovers in *Salix* species, involving movements both within the same chromosome and between nonhomologous chromosomes, indicates the repeated involvement of pericentromeric genome regions that already recombined very infrequently, rather than new nonrecombining regions evolving after a sex-determining gene appeared. Pericentromeric regions may be particularly likely to undergo rearrangements, such as the inversions observed here, and deletions, and these locations may also explain the expanded X-linked regions just mentioned. As discussed in Wang et al. (2024a), similar processes are likely to be involved in the evolution of sex-linked regions in the platyfish, *Xiphophorus maculatus* (Volff and Schartl 2001). Pericentromeric regions can now be studied, despite their high repetitive content, now that long-read sequencing is possible, making it possible to test these ideas in the future.

## Materials and Methods

### Plant Material

Young leaf, catkin, stem, and root samples of male *S. mesnyi* (Samehe19M) and male *S. triandra* (HL00105) were collected, frozen in liquid nitrogen, and stored at −80 °C for genome and RNA sequencing. We collected 41 individuals (21 females and 20 males) of *S. mesnyi* from four wild populations and Shanghai Chenshan Botanical Garden. The leaves of each individual were dried with silica gel for genome re-sequencing. We also downloaded short re-sequencing reads from 20 females and 19 males of *S. triandra* obtained by Wang et al. (2023) to distinguish the 15X and 15Y haplotypes in this species. Supplementary tables S24 and S25, Supplementary Material online describes all details of the plant material used.

### Library Construction and Genome Sequencing

Young leaves of Samehe19M and HL00105 materials were used for genome sequencing. For Illumina PCR-free sequencing, a total genomic DNA was extracted from the two samples using a Qiagen DNeasy Plant Mini Kit (Qiagen). Sequencing libraries were generated using the Illumina TruSeq DNA PCR-Free Library Preparation Kit (Illumina). After quality assessment using an Agilent Bioanalyzer 2100 system (Agilent), the libraries were sequenced on an Illumina NovaSeq 6000 platform by Beijing Novogene Bioinformatics Technology (hereinafter referred to as Novogene). For Hi-C and HiFi sequencing, total genomic DNA was extracted by the CTAB method (Chang et al. 1993). For Hi-C sequencing, fresh leaves from the two samples were preserved in a 4% formaldehyde solution in MS buffer to facilitate fixation. Following this, crosslinked DNA was isolated from the nuclei. To prepare the DNA for sequencing, the DpnII restriction enzyme was employed for digestion, and the resulting fragments were labeled with biotin, purified, ligated, and subsequently sequenced. The Hi-C libraries were sequenced by Novogene on an Illumina Hiseq X Ten platform. For the single-molecule real-time sequencing, HiFi libraries were prepared using the SMRTbell Express Template Prep Kit 2.0 (PacBio), featuring an insert size of 15 kb. DNA fragmentation was achieved using the Diagenode Megaruptor system, followed by concentration utilizing AMPure PB Beads from PacBio (CA, USA). The size selection of libraries was performed using the BluePippin System, and the sequencing process was carried out by Novogene on the PacBio Sequel II platform.

### Genome Assembly

We used the same assembly method for both species, first using the CCS software (https://github.com/PacificBiosciences/ccs) to generate accurate PacBio HiFi reads. These reads were employed for the initial contig assembly using the Hifiasm pipeline v.0.16-r375 (Cheng et al. 2021) with default settings. We filtered the Hi-C reads using Fastp v.0.23.2 (Chen et al. 2018). Juicer v.1.5.6 (Durand et al. 2016) and 3d-DNA pipeline v180922 (Dudchenko et al. 2017) were then employed to yield comprehensive chromosome assemblies based on the filtered Hi-C reads. LR_Gapcloser v1.1 (Xu et al. 2019) and Minimap2 (Li 2018) were applied to improve the continuity of the assemblies using the HiFi reads. We also utilized NextPolish v.1.41 (Hu et al. 2020) to improve the accuracy of the assemblies’ base content by incorporating Illumina short reads. GetOrganelle v.1.7.5 (Jin et al. 2020) was used to assemble the chloroplast and mitochondrial genomes. Finally, BUSCO (v. 5.3.2; http://busco.ezlab.org/) analysis was conducted to evaluate the genome assemblies’ completeness, using embryophyta_odb10 database. The chromosomes were numbered chr01*a*-chr19*a* and chr01*b*-chr19*b* (*a* and *b* represent two haplotypes), following the 19 chromosomes arrangement of *S. arbutifolia* (Wang et al. 2024a).

### Annotation of Genes and Repetitive Sequences

We used EDTA to identify transposable elements, producing a TE library (Ou et al. 2019). Then, RepeatMasker (http://www.repeatmasker.org/RepeatMasker) was used to identify repetitive regions in the genomes. We used the MAKER (Cantarel et al. 2008) annotation pipeline for gene annotation of repeat masked assemblies, combining evidence from transcriptome data, protein homology-based, and ab initio prediction. AUGUSTUS v.3.4.0 (Stanke et al. 2008) was used for ab initio prediction of protein-coding genes. For homology-based gene annotation, the protein sequences from published data from 17 species of Salicaceae and *Arabidopsis thaliana* (supplementary table S26, Supplementary Material online) (a total of 278,011 protein sequences) were aligned to the genomes using BLASTX. For transcriptome-based prediction, we used three strategies for transcriptome assembly. We conducted de novo assembly initially using Trinity v.2.0.6 (Grabherr et al. 2011), and then, after aligning mRNA reads to the genomes with HISAT2 v.2.1.0 (Kim et al. 2015), we separately used Trinity's genome-guided mode and StringTie v.2.1.5 (Pertea et al. 2015). We then used PASA pipeline for gene structure annotation. Finally, EvidenceModeler (Haas et al. 2008) was used to generate consistent gene annotations. Functional annotation of the predicted genes was done by aligning with the Swiss_Prot, TrEMBL, NR, *A. thaliana*, and InterPro protein databases using Diamond v.0.9.24 (Buchfink et al. 2015). Gene ontology and KEGG were also assigned by eggNOG-Mapper (Huerta-Cepas et al. 2017). Annotation of tRNA, rRNA sequences, and other noncoding RNAs followed the method outlined in He et al. (2021).

### Determining the Sex-linked Regions

Total genomic DNA was extracted from leaves of *S. mesnyi* using the Qiagen DNeasy Plant Mini Kit (Qiagen, Valencia, CA, USA) following the manufacturer instructions. Whole genome re-sequencing using paired-end libraries were performed on Novogene. We filtered the low-quality raw reads and removed adapters using Fastp v.0.23.2 with parameters “-cut_tail -n_base_limit 3 -length_required 60 -correction” and used clean reads for the CQ and F_ST_ analyses described below.

To identify the X and Y haplotypes containing respectively the X and Y-linked regions in *S. mesnyi* and *S. triandra*, we employed the CQ method, utilizing CQ-calculate.pl (Hall et al. 2013) following the approach of He et al. (2021). Clean reads from individuals of known sexes (41 of *S. mesnyi* and 39 of *S. triandra*, see above) were mapped to haplotypes *a* and *b* separately, and then coverage was calculated in 50-kb nonoverlapping windows. In an XY system, where X- and Y-linked sequences differ, or where many sequences are absent from the Y chromosome, coverage ratios of female to male sequences (CQ values) should be approximately two when the X haplotype is used as the reference, versus close to 1 and zero, respectively, for autosomal and Y-linked windows. If *S. mesnyi* had a 7XY system like other species in the *Salix* clade, we would expect to find a chromosome 7 region with CQ values close to 0. Similarly, the chromosome 15Y in *S. triandra*, which has a 15XY system, should carry a region with CQ close to 0. Regions with Y-specific sequence due to insertions of sequence into the Y can also potentially be detected.

The reads were aligned to the genome assemblies using the BWA-MEM algorithm of BWA 0.7.12 (Li and Durbin 2009), and GATK v. 4.1.8.1 and VCFtools (Danecek et al. 2011) were employed to filter and select high-quality SNPs. We excluded (i) sites with coverage above twice the mean depth at variant sites across all samples, (ii) nonbiallelic SNPs, and (iii) SNPs with >10% missing information and/or minor allele frequency <5%.

Weighted F_ST_ values between the sexes were calculated in 100-kb overlapping windows with 10-kb steps using Weir and Cockerham (1984) estimator based on SNP datasets with VCFtools. A changepoint package (Killick and Eckley 2014) was used to detect regions where the F_ST_ values change significantly, in order to detect candidate SLRs (He et al. 2021).

We used LDBlockShow v.1.36 (Dong et al. 2021) to calculate and visualize LD patterns between SNPs for the 7X, 7Y, 15*a*, and 15*b* of *S. mesnyi*, as well as 7*a*, 7*b*, 15X, and 15Y of *S. triandra*. PopLDdecay (Zhang et al. 2019) was used to estimate LD decay for the autosomes (including PARs), X-SLRs, and Y-SLRs of *S. mesnyi* and *S. triandra*. We identified possible pericentromeric regions in the *S. mesnyi* and *S. triandra* genomes based on the observed high density of TEs and low gene content, which are expected in pericentromeric regions (Charlesworth et al. 1994), as well as higher LD.

### Identification of X and Y-SLR Specific Genes

X-SLR genes with no BLAST hits in the Y-SLR were classified as X-SLR specific, and vice versa for Y-SLR specific genes. To further classify genes specific to the X- and Y-SLRs in 7XY and 15XY system willow species, we used the 7*a* haplotype of the autosomal chromosome 7 of *S. arbutifolia* (15XY) as an outgroup for the 7XY species, and the autosomal 15*a* haplotype of *S. dunnii* (7XY) for the 15XY species. These outgroups represent the likely ancestral states of the relevant sex chromosomes (Wang et al. 2024a). We used the following criteria to detect gene insertions and losses: if a gene specific to the X- or Y-SLR has a homolog in the inferred ancestral chromosome, it was classified as a gene loss from the Y or X, as appropriate; if no BLAST hit was detected, it was classified as a gene insertion in the Y or X. We estimated degeneration of the regions inferred to be SLRs, using the numbers of gene losses expressed as proportions of the total number of genes shared between the X and Y haplotypes in the SLR. We used Y-specific genes identified in the 7XY and 15XY systems to identify genes shared by the Y chromosomes of different species, using BLASTP.

### Identifying Intact *ARR17*-like Genes and Partial Duplicates, and *MSF* Sequences

To identify *ARR17*-like sequences, we used BLASTN to search the whole genomes of *S. mesnyi* and *S. triandra* with the *Populus* species sequence Potri.019G133600 (Muller et al. 2020) as the query. The intact *ARR17*-like copy includes five protein-coding exons. We categorized duplicates lacking any of these exons in the BLASTN results as partial sequences (Wang et al. 2022). To identify counterparts of *MSF* (a shared gene on Y chromosomes) and identify their locations in willow genomes, we used *MSF* exons as queries in BLASTN searches using the whole genome assemblies of species with phased SLRs. We also searched for homologs of the PISTILLATA (*PI*) gene, a B-class gene in the ABCDE flower development model (Lamb and Irish 2003), that is essential for stamen development and is indirectly regulated by an intact *ARR17*-like gene (Hou et al. 2022; Leite Montalvao et al. 2022). We used BLASTN to search the whole genomes of *S. mesnyi* and *S. triandra* using the *PI* homolog in the *P. trichocarpa*, Potri.002G079000 as the query.

### Synteny and Phylogenetic Analysis

To study chromosomal rearrangements in the sex chromosomes and their corresponding homologous autosomes in *Salix* species, we did separate synteny analyses in the two clades, analyzing the sex chromosomes and their homologous autosomes, as well as comparing homologous autosomes; the analyses used protein-coding genes and the Python version of MCScan (Tang et al. 2008). The same method was used for synteny analysis between chromosomes 13 and 19 of *S. mesnyi*, *S. triandra*, and *P. trichocarpa*, and chromosomes 7 and 12 of *I. polycarpa*, to determine paralogs created by the shared WGD event described above.

We used OrthoFinder (Emms and Kelly 2019) to detect SCOs in *Salix* species whose genomes have been assembled (*S. mesnyi*, *S. dunnii*, *S. chaenomeloides*, *S. triandra*, *S. arbutifolia*, *S. polyclona*, *S. viminalis* (Almeida et al. 2020), *S. suchowensis* (Dai et al. 2014), and *S. purpurea*) and the two outgroup species (*P. trichocarpa* and *I. polycarpa*) for phylogenetic reconstruction. The OrthoFinder results were also used to detect SCOs within the SLRs of species with each of the three sex determination systems (7XY, 15XY, and 15ZW) in the *Salix* genus; this was done separately for *Salix* clade species (*S. mesnyi*, *S. dunnii*, and *S. chaenomeloides*) and *Vetrix* clade species (*S. triandra*, *S. arbutifolia*, *S. polyclona*, and *S. purpurea*). Since gametologs start diverging as soon as recombination stops, SLRs in which recombination was suppressed at different times, relative to the split of related species, should exhibit different topologies, indicating the relative timing (Handley et al. 2004; Bergero and Charlesworth 2009; Zhang et al. 2022). We also estimated phylogenetic trees for two Y-linked sex-determining candidates, including the exon 1 sequences of the intact and partial *ARR17*-like duplicates, and the *MSF* sequences together with their chromosome 13 counterparts, as well as counterparts from both outgroup species. MAGUS software (Smirnov and Warnow 2021) was used to align sequences, and IQ-TREE (Nguyen et al. 2015) to estimate gene trees. Finally, ASTRAL v. 5.7.8 (Zhang et al. 2018) was employed to infer species trees from the gene trees.

### Expression of *MSF*, *PI*, and *ARR17*-like Sequences

To study the expression patterns of *MSF* and *ARR17*-like genes, buds, and catkins were collected from male (HL00105) and female (HL00104) *S. triandra* individuals. For each sex and individual, estimates were obtained from three biological replicates. Total RNA of buds and catkins were extracted using the Plant RNA Purification Reagent (Invitrogen). Transcriptome libraries for RNA-seq were constructed utilizing the TruSeq RNA sample preparation kit (Illumina), sequenced on an Illumina Novaseq 6000 platform by Novogene. Additionally, transcriptome data were downloaded for six stages of male and female bud of *S. dunnii* (T1-T5: flower bud stage; T6: anthesis onset) from BioProject accession number PRJNA1110836, and data (designated T7) for mature male and female catkins of *S. dunnii* from He et al. (2024). The RNA-seq reads of *S. triandra* and *S. dunnii* were mapped to their own reference genomes using HISAT2 v2.1.0 (Kim et al. 2015), and counts were assigned using featureCounts v.2.0.1 (Liao et al. 2014). To detect sRNAs produced by the *ARR17*-like partial duplicates on the 15Y chromosome of *S. triandra*, we performed sRNA sequencing. Total RNA from male (HL00105) buds of *S. triandra* was extracted using RNAprep Pure Plant Plus Kit (Tiangen, China), with three biological replicates. The sRNA with adapters at both ends was hybridized with the reverse transcription primers to synthesize the first-strand cDNA. PCR enrichment was used to obtain the double-stranded cDNA library. After purification, a library with an insert size of 18 to 40 bp was selected for sequencing. Small RNA-seq libraries were sequenced on an Illumina NovaSeq 6000 platform, and then sRNAminer v1.1.2 (Li et al. 2024) was used to analyze the distribution of sRNAs near the exon 1 of *ARR17*-like partial duplicates.

## Supplementary Material

msae235_Supplementary_Data

## Data Availability

Sequencing data for genome assembly and annotation and transcriptome data for *Salix mesnyi* and *Salix triandra* can be downloaded from the National Center for Biotechnology Information (NCBI) under the BioProject accession PRJNA1110836. The genome assemblies of *Salix mesnyi* and *Salix triandra* can be downloaded from the National Genomics Data Center (NGDC) under BioProject accessions PRJCA016000 and PRJCA025661, respectively.

## References

[msae235-B1] Akagi T, Pilkington SM, Varkonyi-Gasic E, Henry IM, Sugano SS, Sonoda M, Firl A, McNeilage MA, Douglas MJ, Wang T, et al Two Y-chromosome-encoded genes determine sex in kiwifruit. Nat Plants. 2019:5(8):801–809. 10.1038/s41477-019-0489-6. 31383971

[msae235-B2] Akagi T, Varkonyi-Gasic E, Shirasawa K, Catanach A, Henry IM, Mertten D, Datson P, Masuda K, Fujita N, Kuwada E, et al Recurrent neo-sex chromosome evolution in kiwifruit. Nat Plants. 2023:9(3):393–402. 10.1038/s41477-023-01361-9. 36879018

[msae235-B3] Almeida P, Proux-Wera E, Churcher A, Soler L, Dainat J, Pucholt P, Nordlund J, Martin T, Ronnberg-Wastljung AC, Nystedt B, et al Genome assembly of the basket willow, *Salix viminalis*, reveals earliest stages of sex chromosome expansion. BMC Biol. 2020:18(1):78. 10.1186/s12915-020-00808-1. 32605573 PMC7329446

[msae235-B4] Bergero R, Charlesworth D. The evolution of restricted recombination in sex chromosomes. Trends Ecol Evol. 2009:24(2):94–102. 10.1016/j.tree.2008.09.010. 19100654

[msae235-B5] Buchfink B, Xie C, Huson DH. Fast and sensitive protein alignment using DIAMOND. Nat Methods. 2015:12(1):59–60. 10.1038/nmeth.3176. 25402007

[msae235-B6] Cantarel BL, Korf I, Robb SMC, Parra G, Ross E, Moore B, Holt C, Alvarado AS, Yandell M. MAKER: An easy-to-use annotation pipeline designed for emerging model organism genomes. Genome Res. 2008:18(1):188–196. 10.1101/gr.6743907. 18025269 PMC2134774

[msae235-B7] Cauret CMS, Mortimer SME, Roberti MC, Ashman T-L, Liston A. Chromosome-scale assembly with a phased sex-determining region resolves features of early Z and W chromosome differentiation in a wild octoploid strawberry. G3 (Bethesda). 2022:12(8):jkac139. 10.1093/g3journal/jkac139. 35666193 PMC9339316

[msae235-B8] Chang S, Puryear J, Cairney J. A simple and efficient method for isolating RNA from pine trees. Plant Mol Biol Report. 1993:11(2):113–116. 10.1007/BF02670468.

[msae235-B9] Charlesworth B, Charlesworth D. A model for the evolution of dioecy and gynodioecy. Am Nat. 1978:112(988):975–997. 10.1086/283342.

[msae235-B10] Charlesworth B, Sniegowski P, Stephan W. The evolutionary dynamics of repetitive DNA in eukaryotes. Nature. 1994:371(6494):215–220. 10.1038/371215a0. 8078581

[msae235-B11] Charlesworth D . Plant sex chromosome evolution. J Exp Bot. 2013:64(2):405–420. 10.1093/jxb/ers322. 23125359

[msae235-B12] Charlesworth D, Bergero R, Graham C, Gardner J, Keegan K. How did the guppy Y chromosome evolve? PLoS Genet. 2021:17(8):e1009704. 10.1371/journal.pgen.1009704. 34370728 PMC8376059

[msae235-B13] Charlesworth D, Harkess A. Why should we study plant sex chromosomes? Plant Cell. 2024:36(5):1242–1256. 10.1093/plcell/koad278. 38163640 PMC11062472

[msae235-B14] Chen S, Zhou Y, Chen Y, Gu J. Fastp: an ultra-fast all-in-one FASTQ preprocessor. Bioinformatics. 2018:34(17):i884–i890. 10.1093/bioinformatics/bty560. 30423086 PMC6129281

[msae235-B15] Cheng H, Concepcion GT, Feng X, Zhang H, Li H. Haplotype-resolved de novo assembly using phased assembly graphs with hifiasm. Nat Methods. 2021:18(2):170–175. 10.1038/s41592-020-01056-5. 33526886 PMC7961889

[msae235-B16] Cronk Q, Soolanayakanahally R, Bräutigam K. Gene expression trajectories during male and female reproductive development in balsam poplar (*Populus balsamifera* L.). Sci Rep. 2020:10(1):8413. 10.1038/s41598-020-64938-w. 32439903 PMC7242425

[msae235-B17] Dai X, Hu Q, Cai Q, Feng K, Ye N, Tuskan GA, Milne R, Chen Y, Wan Z, Wang Z, et al The willow genome and divergent evolution from poplar after the common genome duplication. Cell Res. 2014:24(10):1274–1277. 10.1038/cr.2014.83. 24980958 PMC4185352

[msae235-B18] Danecek P, Auton A, Abecasis G, Albers CA, Banks E, DePristo MA, Handsaker RE, Lunter G, Marth GT, Sherry ST, et al The variant call format and VCFtools. Bioinformatics. 2011:27(15):2156–2158. 10.1093/bioinformatics/btr330. 21653522 PMC3137218

[msae235-B19] Darwin CR . The different forms of flowers on plants of the same species. London: J. Murray; 1877.

[msae235-B20] Dong S-S, He W-M, Ji J-J, Zhang C, Guo Y, Yang T-L. LDBlockShow: a fast and convenient tool for visualizing linkage disequilibrium and haplotype blocks based on variant call format files. Brief Bioinform. 2021:22(4):bbaa227. 10.1093/bib/bbaa227. 33126247

[msae235-B21] Dudchenko O, Batra SS, Omer AD, Nyquist SK, Hoeger M, Durand NC, Shamim MS, Machol I, Lander ES, Aiden AP, et al De novo assembly of the *Aedes aegypti* genome using Hi-C yields chromosome-length scaffolds. Science. 2017:356(6333):92–95. 10.1126/science.aal3327. 28336562 PMC5635820

[msae235-B22] Durand NC, Shamim MS, Machol I, Rao SSP, Huntley MH, Lander ES, Aiden EL. Juicer provides a one-click system for analyzing loop-resolution Hi-C experiments. Cell Syst. 2016:3(1):95–98. 10.1016/j.cels.2016.07.002. 27467249 PMC5846465

[msae235-B23] Emms DM, Kelly S. OrthoFinder: phylogenetic orthology inference for comparative genomics. Genome Biol. 2019:20(1):238. 10.1186/s13059-019-1832-y. 31727128 PMC6857279

[msae235-B24] Gladysh NS, Kovalev MA, Lantsova MS, Popchenko MI, Bolsheva NL, Starkova AM, Bulavkina EV, Karpov DS, Kudryavtsev AA, Kudryavtseva AV. The molecular and genetic mechanisms of sex determination in poplar. Molecular Biology. 2024:58(2):178–191. 10.1134/S0026893324020067. 39355879

[msae235-B25] Goldberg MT, Spigler RB, Ashman T-L. Comparative genetic mapping points to different sex chromosomes in sibling species of wild strawberry (*Fragaria*). Genetics. 2010:186(4):1425–1433. 10.1534/genetics.110.122911. 20923978 PMC2998321

[msae235-B26] Grabherr MG, Haas BJ, Yassour M, Levin JZ, Thompson DA, Amit I, Adiconis X, Fan L, Raychowdhury R, Zeng Q, et al Full-length transcriptome assembly from RNA-Seq data without a reference genome. Nat Biotechnol. 2011:29(7):644–652. 10.1038/nbt.1883. 21572440 PMC3571712

[msae235-B27] Gulyaev S, Cai X-J, Guo F-Y, Kikuchi S, Applequist WL, Zhang Z-X, Horandl E, He L. The phylogeny of *Salix* revealed by whole genome re-sequencing suggests different sex-determination systems in major groups of the genus. Ann Bot. 2022:129(4):485–498. 10.1093/aob/mcac012. 35134824 PMC8944726

[msae235-B28] Haas BJ, Salzberg SL, Zhu W, Pertea M, Allen JE, Orvis J, White O, Buell CR, Wortman JR. Automated eukaryotic gene structure annotation using EVidenceModeler and the Program to Assemble Spliced Alignments. Genome Biol. 2008:9(1):R7. 10.1186/gb-2008-9-1-r7. 18190707 PMC2395244

[msae235-B29] Hall AB, Qi Y, Timoshevskiy V, Sharakhova MV, Sharakhov IV, Tu Z. Six novel Y chromosome genes in anopheles mosquitoes discovered by independently sequencing males and females. BMC Genomics. 2013:14:273. 10.1186/1471-2164-14-273. 23617698 PMC3660176

[msae235-B30] Handley L-J, Ceplitis H, Ellegren H. Evolutionary strata on the chicken Z chromosome: implications for sex chromosome evolution. Genetics. 2004:167(1):367–376. 10.1534/genetics.167.1.367. 15166161 PMC1470863

[msae235-B31] He L, Jia K-H, Zhang R-G, Wang Y, Shi T-L, Li Z-C, Zeng S-W, Cai X-J, Wagner ND, Horandl E, et al Chromosome-scale assembly of the genome of *Salix dunnii* reveals a male-heterogametic sex determination system on chromosome 7. Mol Ecol Resour. 2021:21(6):1966–1982. 10.1111/1755-0998.13362. 33609314 PMC8359994

[msae235-B32] He L, Wang Y, Wang Y, Zhang R-G, Wang Y, Hörandl E, Ma T, Mao Y-F, Mank JE, Ming R. Allopolyploidization from two dioecious ancestors leads to recurrent evolution of sex chromosomes. Nat Commun. 2024:15(1):6893. 10.1038/s41467-024-51158-3. PMC11319354 39134553

[msae235-B33] Hou X-J, Ye L-X, Ai X-Y, Hu C-G, Cheng Z-P, Zhang J-Z. Functional analysis of a PISTILLATA-like gene CcMADS20 involved in floral organs specification in citrus. Plant Sci. 2022:319:111263. 10.1016/j.plantsci.2022.111263. 35487669

[msae235-B34] Hu J, Fan J, Sun Z, Liu S. NextPolish: a fast and efficient genome polishing tool for long-read assembly. Bioinformatics. 2020:36(7):2253–2255. 10.1093/bioinformatics/btz891. 31778144

[msae235-B35] Huerta-Cepas J, Forslund K, Coelho LP, Szklarczyk D, Jensen LJ, von Mering C, Bork P. Fast genome-wide functional annotation through orthology assignment by eggNOG-mapper. Mol Biol Evol. 2017:34(8):2115–2122. 10.1093/molbev/msx148. 28460117 PMC5850834

[msae235-B36] Hyden B, Zou J, Wilkerson DG, Carlson CH, Robles AR, DiFazio SP, Smart LB. Structural variation of a sex-linked region confers monoecy and implicates GATA15 as a master regulator of sex in *Salix purpurea*. New Phytol. 2023:238(6):2512–2523. 10.1111/nph.18853. 36866707

[msae235-B37] Jin J-J, Yu W-B, Yang J-B, Song Y, dePamphilis CW, Yi T-S, Li D-Z. GetOrganelle: a fast and versatile toolkit for accurate de novo assembly of organelle genomes. Genome Biol. 2020:21(1):241. 10.1186/s13059-020-02154-5. 32912315 PMC7488116

[msae235-B38] Killick R, Eckley IA. Changepoint: an R package for changepoint analysis. J Stat Softw. 2014:58(3):1–19. 10.18637/jss.v058.i03.

[msae235-B39] Kim D, Langmead B, Salzberg SL. HISAT: a fast spliced aligner with low memory requirements. Nat Methods. 2015:12(4):357–360. 10.1038/nmeth.3317. 25751142 PMC4655817

[msae235-B40] Lamb RS, Irish VF. Functional divergence within the APETALA3/PISTILLATA floral homeotic gene lineages. Proc Natl Acad Sci U S A. 2003:100(11):6558–6563. 10.1073/pnas.0631708100. 12746493 PMC164485

[msae235-B41] Leite Montalvao AP, Kersten B, Kim G, Fladung M, Muller NA. ARR17 controls dioecy in Populus by repressing B-class MADS-box gene expression. Philos Trans R Soc Lond B Biol Sci. 2022:377(1850):20210217. 10.1098/rstb.2021.0217. 35306887 PMC8935312

[msae235-B42] Levan A, Fredga K, Sandberg AA. Nomenclature for centromeric position on chromosomes. Hereditas. 1964:52(2):201–220. 10.1111/j.1601-5223.1964.tb01953.x.

[msae235-B43] Li G, Chen C, Chen P, Meyers BC, Xia R. sRNAminer: a multifunctional toolkit for next-generation sequencing small RNA data mining in plants. Sci Bull (Beijing). 2024:69(6):784–791. 10.1016/j.scib.2023.12.049. 38246798

[msae235-B44] Li H . Minimap2: pairwise alignment for nucleotide sequences. Bioinformatics. 2018:34(18):3094–3100. 10.1093/bioinformatics/bty191. 29750242 PMC6137996

[msae235-B45] Li H, Durbin R. Fast and accurate short read alignment with Burrows-Wheeler transform. Bioinformatics. 2009:25(14):1754–1760. 10.1093/bioinformatics/btp324. 19451168 PMC2705234

[msae235-B46] Li X, Gao X, Wei Y, Deng L, Ouyang Y, Chen G, Li X, Zhang Q, Wu C. Rice APOPTOSIS INHIBITOR5 coupled with two DEAD-box adenosine 5′-triphosphate-dependent RNA helicases regulates tapetum degeneration. Plant Cell. 2011:23(4):1416–1434. 10.1105/tpc.110.082636. 21467577 PMC3101562

[msae235-B47] Liao Y, Smyth GK, Shi W. featureCounts: an efficient general purpose program for assigning sequence reads to genomic features. Bioinformatics. 2014:30(7):923–930. 10.1093/bioinformatics/btt656. 24227677

[msae235-B48] Ma T, Wang J, Zhou G, Yue Z, Hu Q, Chen Y, Liu B, Qiu Q, Wang Z, Zhang J, et al Genomic insights into salt adaptation in a desert poplar. Nat Commun. 2013:4:2797. 10.1038/ncomms3797. 24256998

[msae235-B49] Ma X, Yu L, Fatima M, Wadlington WH, Hulse-Kemp AM, Zhang X, Zhang S, Xu X, Wang J, Huang H, et al The spinach YY genome reveals sex chromosome evolution, domestication, and introgression history of the species. Genome Biol. 2022:23(1):75. 10.1186/s13059-022-02633-x. 35255946 PMC8902716

[msae235-B50] Meisel RP, Gonzales CA, Luu H. The house fly Y chromosome is young and minimally differentiated from its ancient X chromosome partner. Genome Res. 2017:27(8):1417–1426. 10.1101/gr.215509.116. 28619849 PMC5538557

[msae235-B51] Meisel RP, Olafson PU, Adhikari K, Guerrero FD, Konganti K, Benoit JB. Sex chromosome evolution in muscid flies. G3 (Bethesda). 2020:10(4):1341–1352. 10.1534/g3.119.400923. 32051221 PMC7144080

[msae235-B52] Ming R, Bendahmane A, Renner SS. Sex chromosomes in land plants. Annu Rev Plant Biol. 2011:62:485–514. 10.1146/annurev-arplant-042110-103914. 21526970

[msae235-B53] Moore RC, Purugganan MD. The evolutionary dynamics of plant duplicate genes. Curr Opin Plant Biol. 2005:8(2):122–128. 10.1016/j.pbi.2004.12.001. 15752990

[msae235-B54] Muller NA, Kersten B, Leite Montalvao AP, Mahler N, Bernhardsson C, Brautigam K, Carracedo Lorenzo Z, Hoenicka H, Kumar V, Mader M, et al A single gene underlies the dynamic evolution of poplar sex determination. Nat Plants. 2020:6(6):630–637. 10.1038/s41477-020-0672-9. 32483326

[msae235-B55] Nguyen L-T, Schmidt HA, von Haeseler A, Minh BQ. IQ-TREE: a fast and effective stochastic algorithm for estimating maximum-likelihood phylogenies. Mol Biol Evol. 2015:32(1):268–274. 10.1093/molbev/msu300. 25371430 PMC4271533

[msae235-B56] Ogutcen E, de Lima Ferreira P, Wagner ND, Marinček P, Leong V, Aubona J, Cavender-Bares G, Michálek J, Schroeder J, Sedio L, et al Phylogenetic insights into the Salicaceae: the evolution of willows and beyond. Mol Phylogenet Evol. 2024:199:108161. 10.1016/j.ympev.2024.108161. 39079595

[msae235-B57] Ou S, Su W, Liao Y, Chougule K, Agda JRA, Hellinga AJ, Lugo CSB, Elliott TA, Ware D, Peterson T, et al Benchmarking transposable element annotation methods for creation of a streamlined, comprehensive pipeline. Genome Biol. 2019:20(1). 10.1186/s13059-019-1905-y. PMC6913007 31843001

[msae235-B58] Pan Q, Kay T, Depince A, Adolfi M, Schartl M, Guiguen Y, Herpin A. Evolution of master sex determiners: TGF-beta signalling pathways at regulatory crossroads. Philos Trans R Soc Lond B Biol Sci. 2021:376(1832):20200091. 10.1098/rstb.2020.0091. 34247498 PMC8273507

[msae235-B59] Pertea M, Pertea GM, Antonescu CM, Chang T-C, Mendell JT, Salzberg SL. StringTie enables improved reconstruction of a transcriptome from RNA-seq reads. Nat Biotechnol. 2015:33(3):290–295. 10.1038/nbt.3122. 25690850 PMC4643835

[msae235-B60] Renner SS . The relative and absolute frequencies of angiosperm sexual systems: dioecy, monoecy, gynodioecy, and an updated online database. Am J Bot. 2014:101(10):1588–1596. 10.3732/ajb.1400196. 25326608

[msae235-B61] Sanderson BJ, Feng G, Hu N, Carlson CH, Smart LB, Keefover-Ring K, Yin T, Ma T, Liu J, DiFazio SP, et al Sex determination through X-Y heterogamety in *Salix nigra*. Heredity (Edinb). 2021:126(4):630–639. 10.1038/s41437-020-00397-3. 33510464 PMC8115673

[msae235-B62] She H, Liu Z, Li S, Xu Z, Zhang H, Cheng F, Wu J, Wang X, Deng C, Charlesworth D, et al Evolution of the spinach sex-linked region within a rarely recombining pericentromeric region. Plant Physiol. 2023:193(2):1263–1280. 10.1093/plphys/kiad389. 37403642

[msae235-B63] Smirnov V, Warnow T. MAGUS: multiple sequence alignment using graph clUStering. Bioinformatics. 2021:37(12):1666–1672. 10.1093/bioinformatics/btaa992. 33252662 PMC8289385

[msae235-B64] Stanke M, Diekhans M, Baertsch R, Haussler D. Using native and syntenically mapped cDNA alignments to improve de novo gene finding. Bioinformatics. 2008:24(5):637–644. 10.1093/bioinformatics/btn013. 18218656

[msae235-B65] Tang H, Bowers JE, Wang X, Ming R, Alam M, Paterson AH. Synteny and collinearity in plant genomes. Science. 2008:320(5875):486–488. 10.1126/science.1153917. 18436778

[msae235-B66] Volff JN, Schartl M. Variability of genetic sex determination in poeciliid fishes. Genetica. 2001:111(1/3):101–110. 10.1023/A:1013795415808. 11841158

[msae235-B67] Wang D, Li Y, Li M, Yang W, Ma X, Zhang L, Wang Y, Feng Y, Zhang Y, Zhou R, et al Repeated turnovers keep sex chromosomes young in willows. Genome Biol. 2022:23(1):200. 10.1186/s13059-022-02769-w. 36151581 PMC9502649

[msae235-B68] Wang Y, Cai X, Zhang Y, Hörandl E, Zhang Z, He L. The male-heterogametic sex determination system on chromosome 15 of *Salix triandra* and *Salix arbutifolia* reveals ancestral male heterogamety and subsequent turnover events in the genus *Salix*. Heredity (Edinb). 2023:130(3):122–134. 10.1038/s41437-022-00586-2. 36593355 PMC9981616

[msae235-B69] Wang Y, Gong G-N, Wang Y, Zhang R-G, Horandl E, Zhang Z-X, Charlesworth D, He L. Gap-free X and Y chromosome assemblies of *Salix arbutifolia* reveal an evolutionary change from male to female heterogamety in willows, without a change in the position of the sex-determining locus. New Phytol. 2024a:242(6):2872–2887. 10.1111/nph.19744. 38581199

[msae235-B70] Wang Y, Weng H, Zeng S, Wang Y, Li Y, He L. Identification and expression analysis of flower development-related MADS-box gene family members in *Salix*. J Fujian Agric Forest Univ (Nat Sci Ed). 2024b:53(3):355–363. 10.13323/j.cnki.j.fafu(nat.sci.).202308011.

[msae235-B71] Wang Y, Xue Z-Q, Zhang R-G, Horandl E, Wang X-R, Mank JE, He L. A novel downstream factor in willows replaces the ancestral sex determining gene. bioRxiv 618180. 10.1101/2024.10.14.618180, 16 October 2024, preprint: not peer reviewed.

[msae235-B72] Weir BS, Cockerham CC. Estimating F-statistics for the analysis of population structure. Evolution. 1984:38(6):1358–1370. 10.1111/j.1558-5646.1984.tb05657.x. 28563791

[msae235-B73] Westergaard M . The mechanism of sex determination in dioecious flowering plants. Adv Genet. 1958:9:217–281. 10.1016/S0065-2660(08)60163-7. 13520443

[msae235-B74] Wu J, Nyman T, Wang D-C, Argus GW, Yang Y-P, Chen J-H. Phylogeny of *Salix* subgenus *Salix* s.l. (Salicaceae): delimitation, biogeography, and reticulate evolution. BMC Evol Biol. 2015:15:31. 10.1186/s12862-015-0311-7. 25886526 PMC4357182

[msae235-B75] Xu G-C, Xu T-J, Zhu R, Zhang Y, Li S-Q, Wang H-W, Li J-T. LR_Gapcloser: a tiling path-based gap closer that uses long reads to complete genome assembly. Gigascience. 2019:8(1):giy157. 10.1093/gigascience/giy157. 30576505 PMC6324547

[msae235-B76] Xue L, Wu H, Chen Y, Li X, Hou J, Lu J, Wei S, Dai X, Olson MS, Liu J, et al Evidences for a role of two Y-specific genes in sex determination in *Populus deltoides*. Nat Commun. 2020:11(1):5893. 10.1038/s41467-020-19559-2. 33208755 PMC7674411

[msae235-B77] Xue Z-Q, Applequist WL, Hörandl E, He L. Sex chromosome turnover plays an important role in the maintenance of barriers to post-speciation introgression in willows. Evol Lett. 2024:8(4):467–477. 10.1093/evlett/qrae013. 39100237 PMC11291624

[msae235-B78] Yang H-W, Akagi T, Kawakatsu T, Tao R. Gene networks orchestrated by MeGI: a single-factor mechanism underlying sex determination in persimmon. Plant J. 2019:98(1):97–111. 10.1111/tpj.14202. 30556936 PMC6850717

[msae235-B79] Zhang C, Dong S-S, Xu J-Y, He W-M, Yang T-L. PopLDdecay: a fast and effective tool for linkage disequilibrium decay analysis based on variant call format files. Bioinformatics. 2019:35(10):1786–1788. 10.1093/bioinformatics/bty875. 30321304

[msae235-B80] Zhang H, Sigeman H, Hansson B. Assessment of phylogenetic approaches to study the timing of recombination cessation on sex chromosomes. J Evol Biol. 2022:35(12):1721–1733. 10.1111/jeb.14068. 35895083 PMC10086819

[msae235-B81] Zhang L, Xi Z, Wang M, Guo X, Ma T. Plastome phylogeny and lineage diversification of Salicaceae with focus on poplars and willows. Ecol Evol. 2018:8(16):7817–7823. 10.1002/ece3.4261. 30250665 PMC6145263

[msae235-B82] Zhou R, Macaya-Sanz D, Carlson CH, Schmutz J, Jenkins JW, Kudrna D, Sharma A, Sandor L, Shu S, Barry K, et al A willow sex chromosome reveals convergent evolution of complex palindromic repeats. Genome Biol. 2020:21(1):38. 10.1186/s13059-020-1952-4. 32059685 PMC7023750

[msae235-B83] Zuo Y, Liu H, Li B, Zhao H, Li X, Chen J, Wang L, Zheng Q, He Y, Zhang J, et al The Idesia polycarpa genome provides insights into its evolution and oil biosynthesis. Cell Rep. 2024:43(3):113909. 10.1016/j.celrep.2024.113909. 38451814

