# Supplementary material for: Evolution of Sex-linked Genes and the Role of Pericentromeric Regions in Sex Chromosomes: Insights from Diploid Willows
Source: Mol Biol Evol. 2024 Nov 12;41(11):msae235. doi: 10.1093/molbev/msae235 (PMC11580687; doi:10.1093/molbev/msae235)

Supplementary Figures

Figure S1

Chromosome quotients (CQ) in 50-kb nonoverlapping windows of haplotype *a* of *Salix mesnyi*, which has a 7XY system.

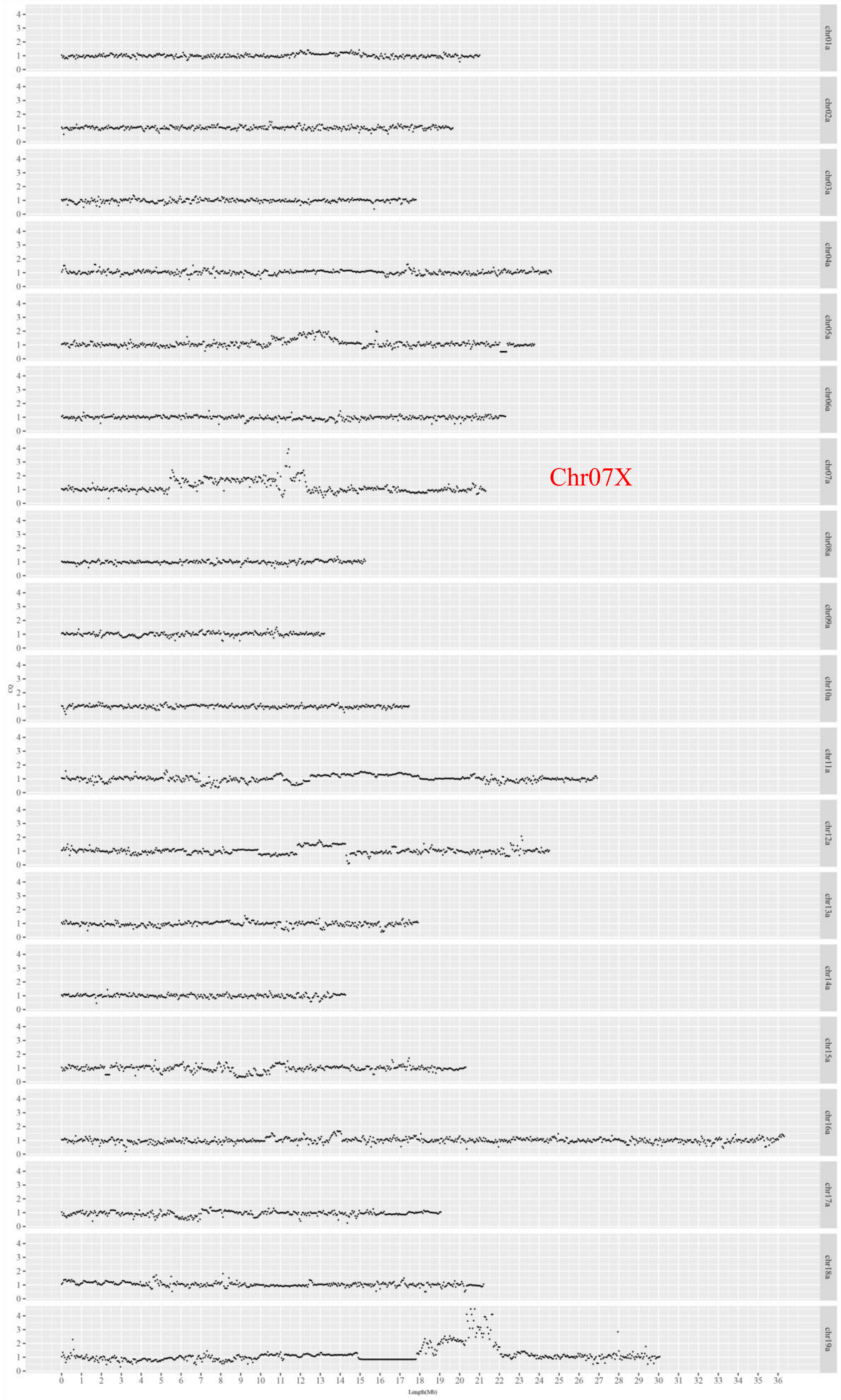

Figure S2

Chromosome quotients (CQ) in 50-kb nonoverlapping windows of haplotype *b* of *Salix mesnyi*, which has a 7XY system.

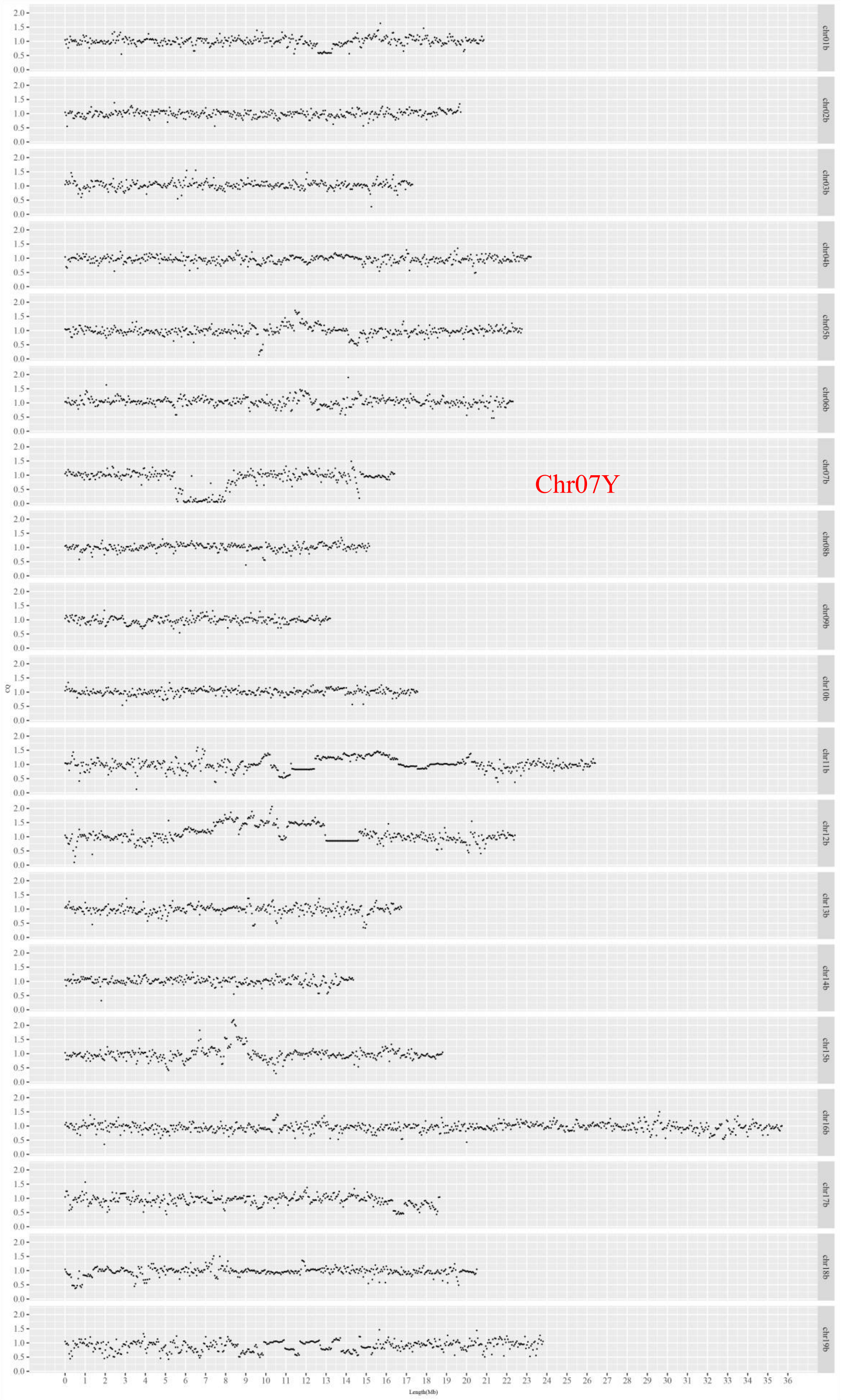

Figure S3

Chromosome quotients (CQ) in 50-kb nonoverlapping windows of haplotype *a* and *b* of *Salix triandra*, which has an 15XY system.

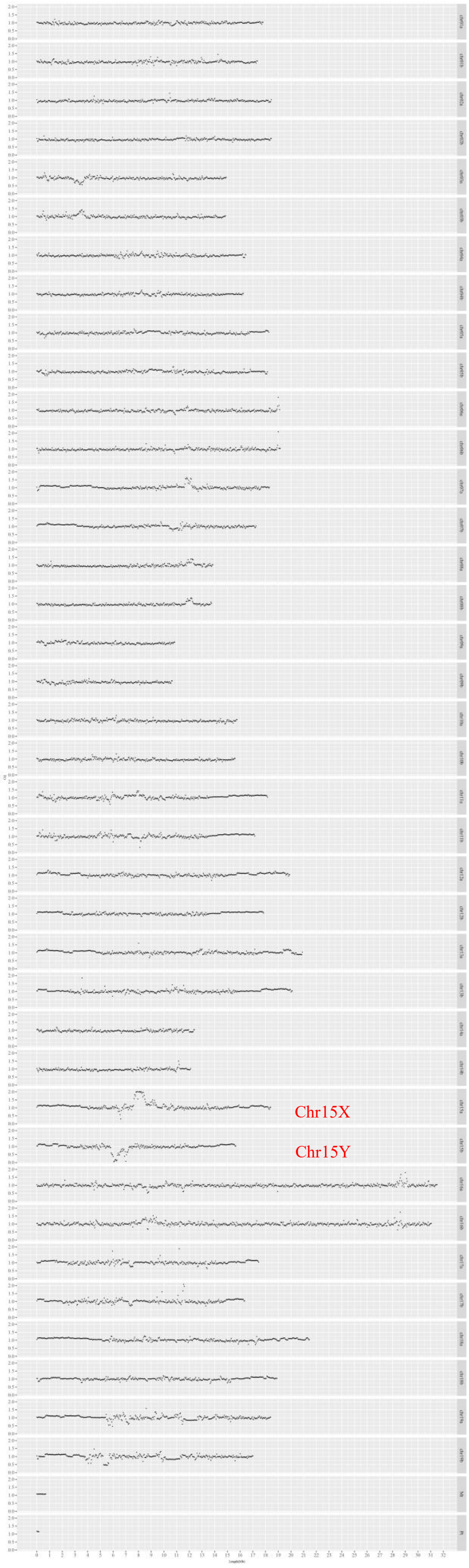

Figure S4

F_{ST} values between the sexes for 100-kb overlapping windows of the *Salix mesnyi* haplotype *a* in 10-kb windows.

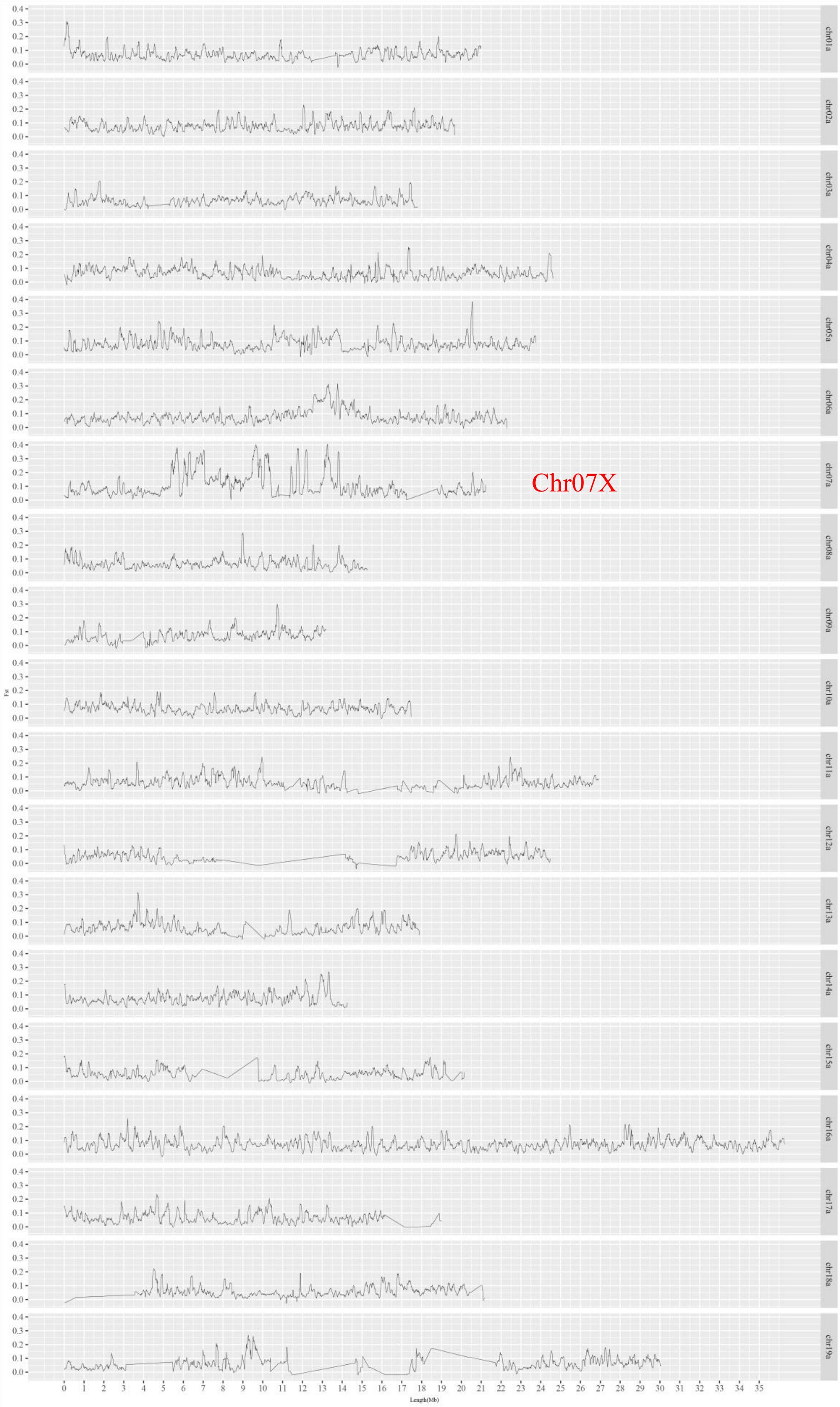

Figure S5

F_{ST} values between the sexes for 100-kb overlapping windows of the *Salix mesnyi* haplotype *b* calculated with 10-kb steps.

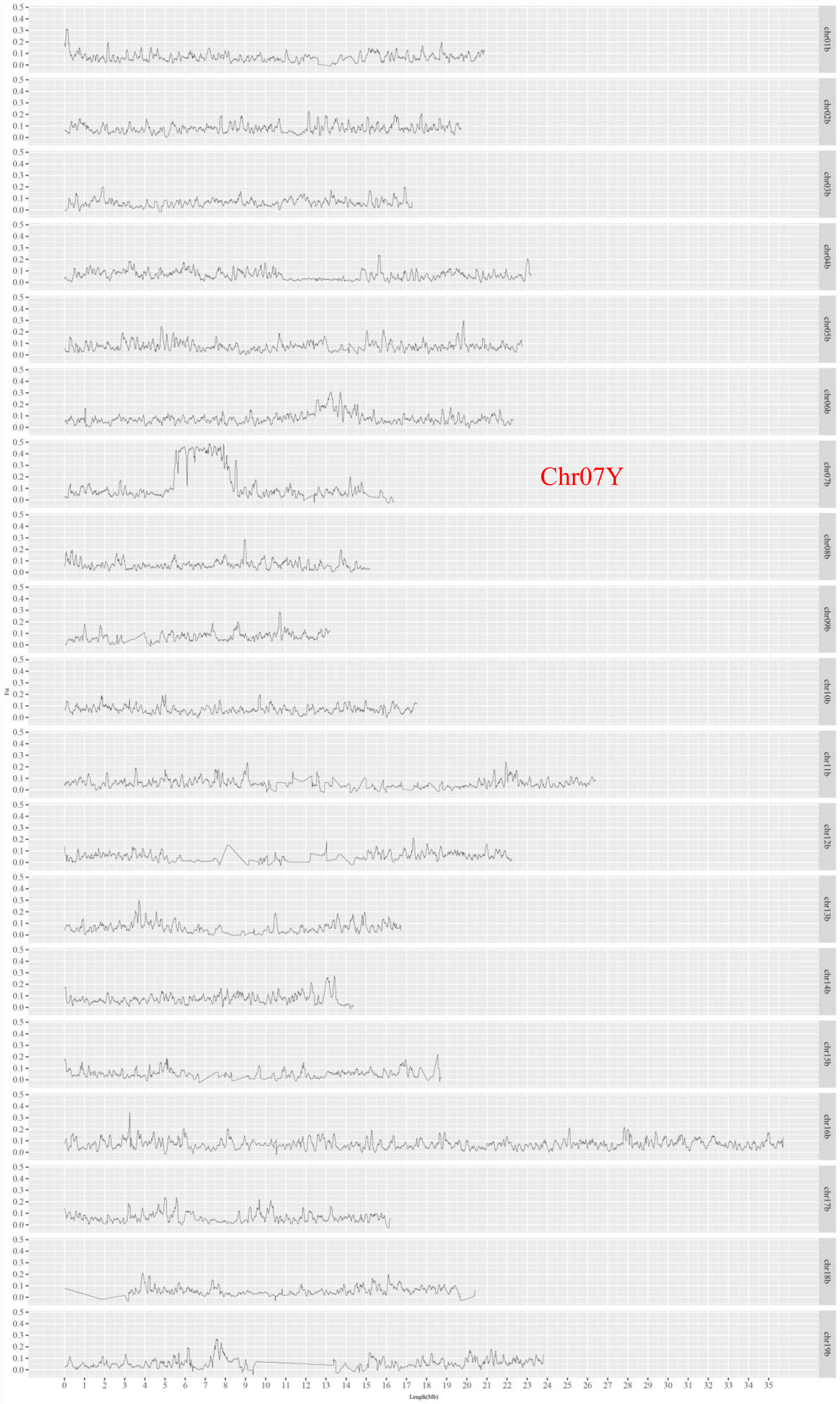

Figure S6

F_{ST} values between the sexes for 100-kb overlapping windows of the *Salix triandra* haplotype *a* calculated with 10-kb steps.

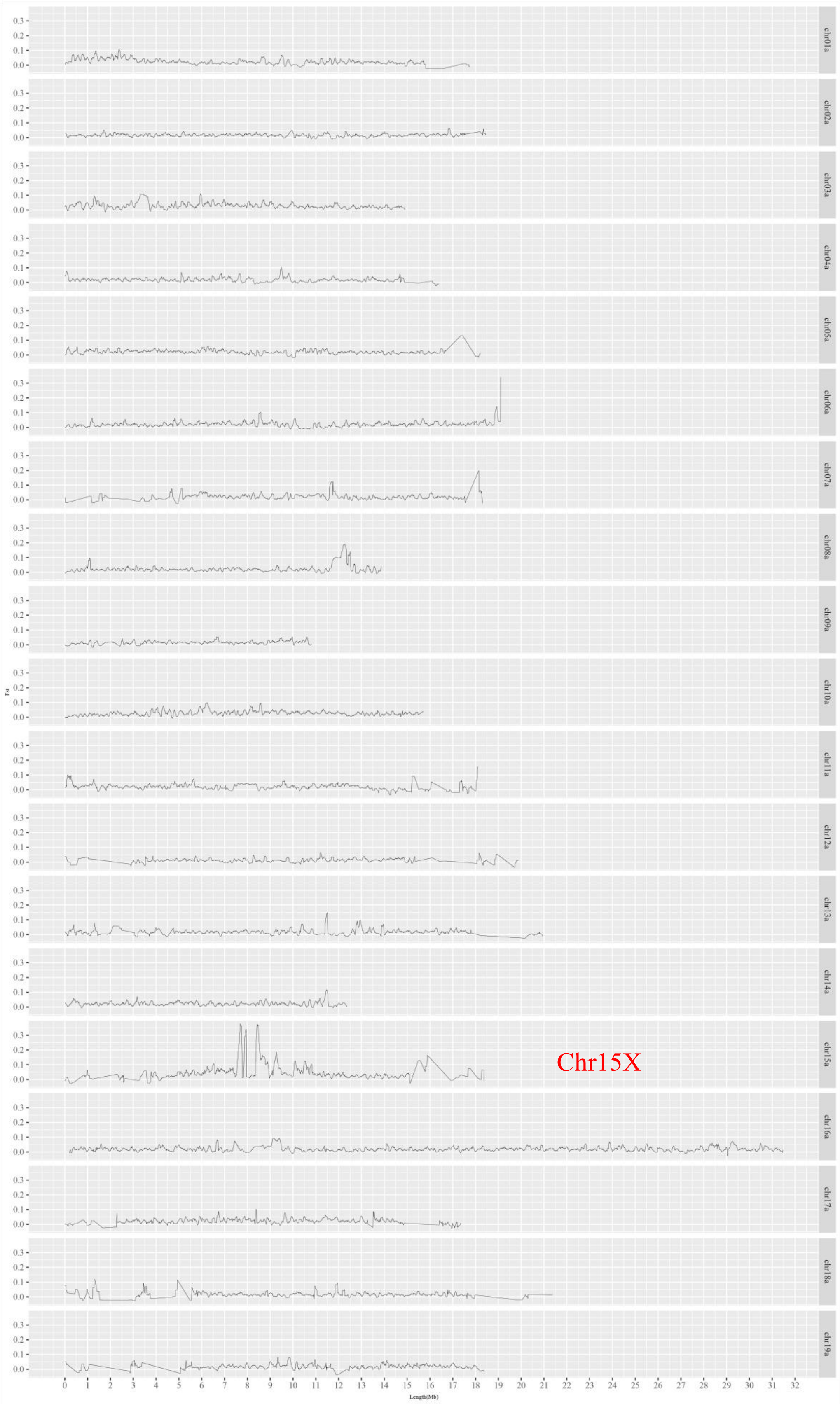

Figure S7

F_{ST} values between the sexes for 100-kb overlapping windows of the *Salix triandra* haplotype *b* calculated with 10-kb steps.

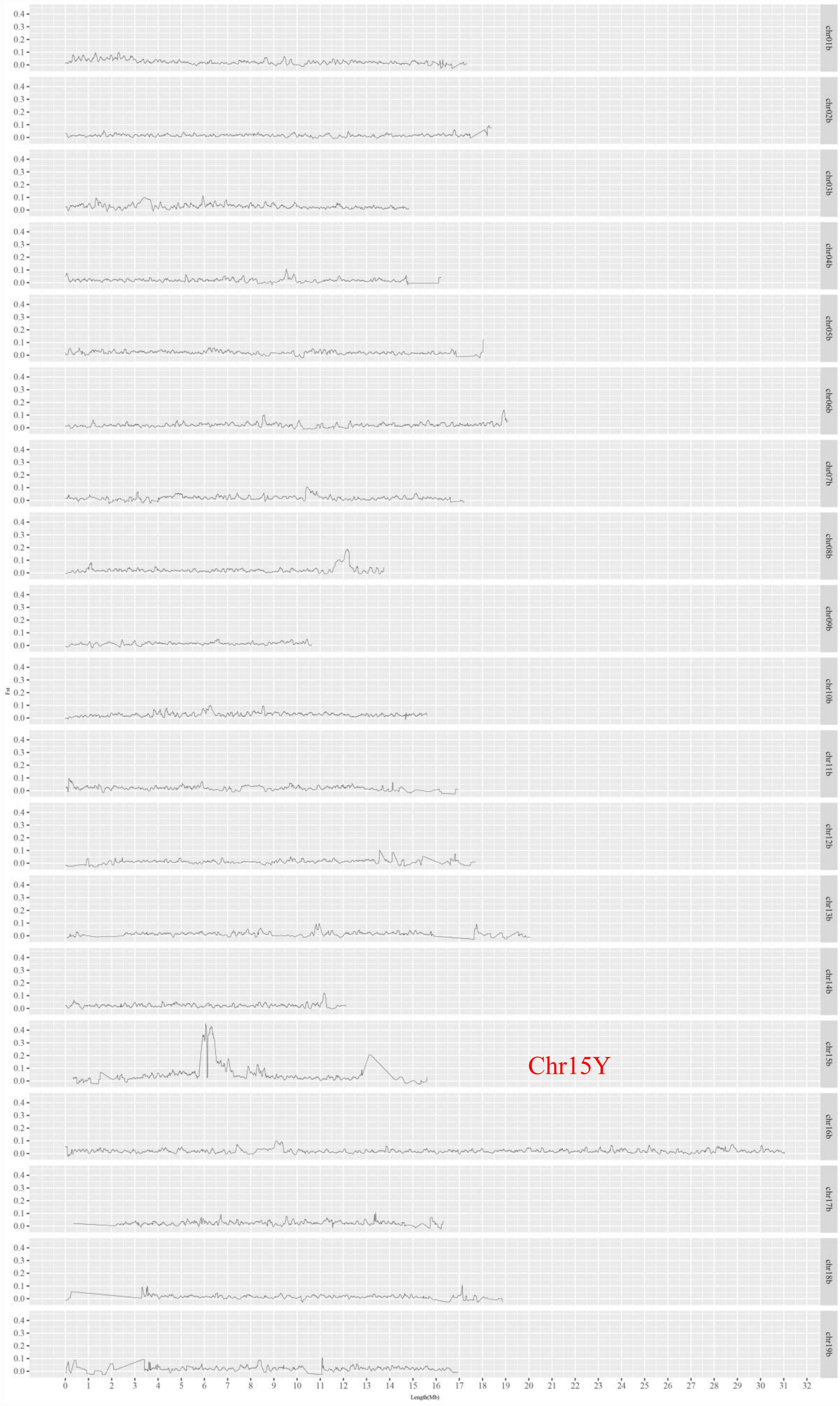

Figure S8

Circos plot of all 38 *Salix mesnyi* chromosomes. a, the chromosome lengths in Mb; b, gene density; c, total TE density; d, LTR-Gypsy density and e, LTR-copia density. Blue regions on each chromosome represent their pericentromeric regions inferred from higher TE and lower gene densities.

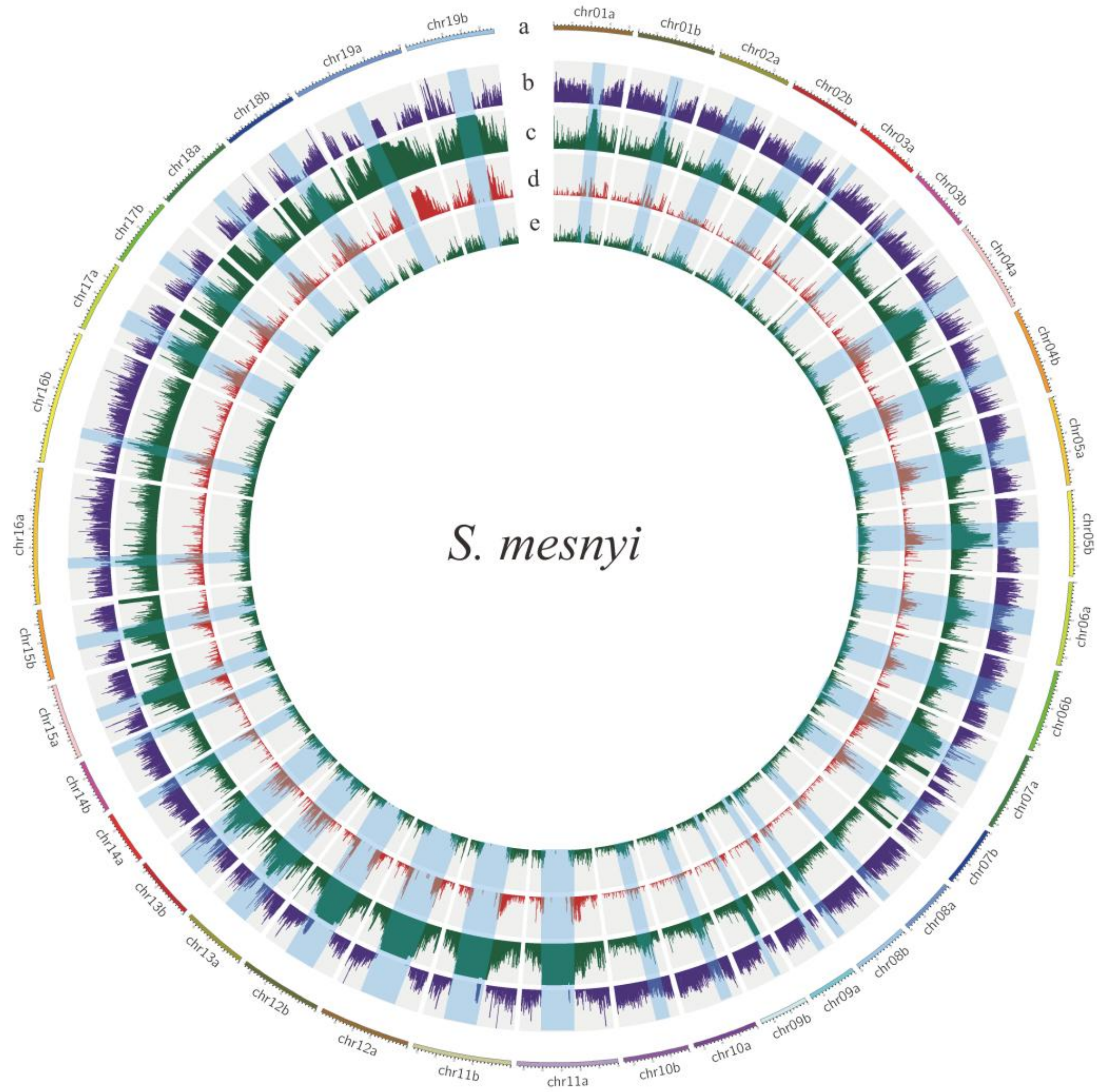

Figure S9

Circos plot of all 38 *Salix triandra* chromosomes. a, the chromosome lengths in Mb; b, gene density; c, total TE density; d, LTR-Gypsy density and e, LTR-copia density. Blue regions on each chromosome represent their pericentromeric regions inferred from the TE densities and gene densities.

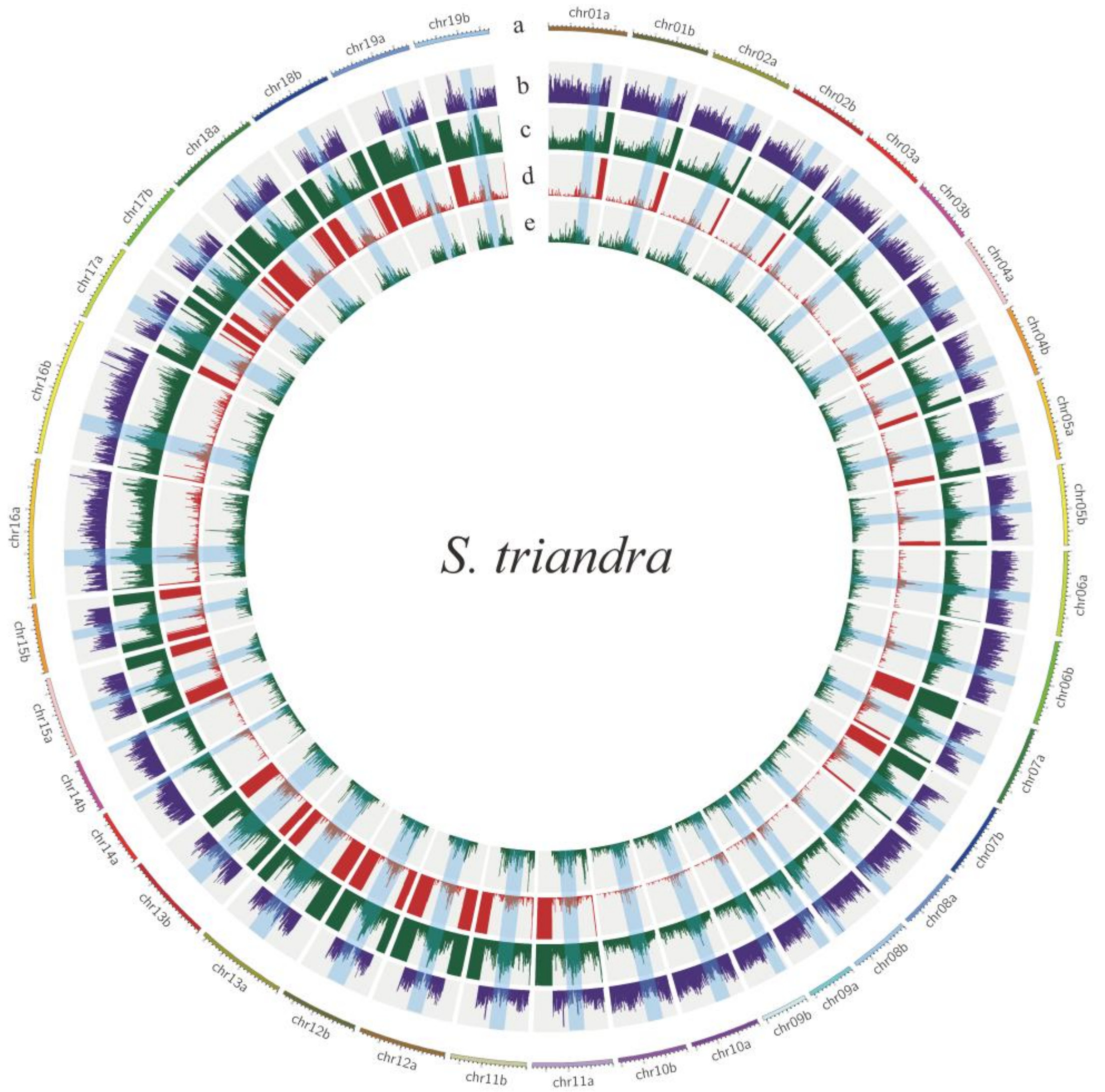

Figure S10

Linkage disequilibrium (LD) decay patterns of autosomes (including PARs), X-SLRs, and Y-SLRs of *Salix mesnyi* (a-c) and *Salix triandra* (d-f). LD is expressed as the squared allele frequency correlation (r^2) between two sites whose distances apart are indicated on the X-axis.

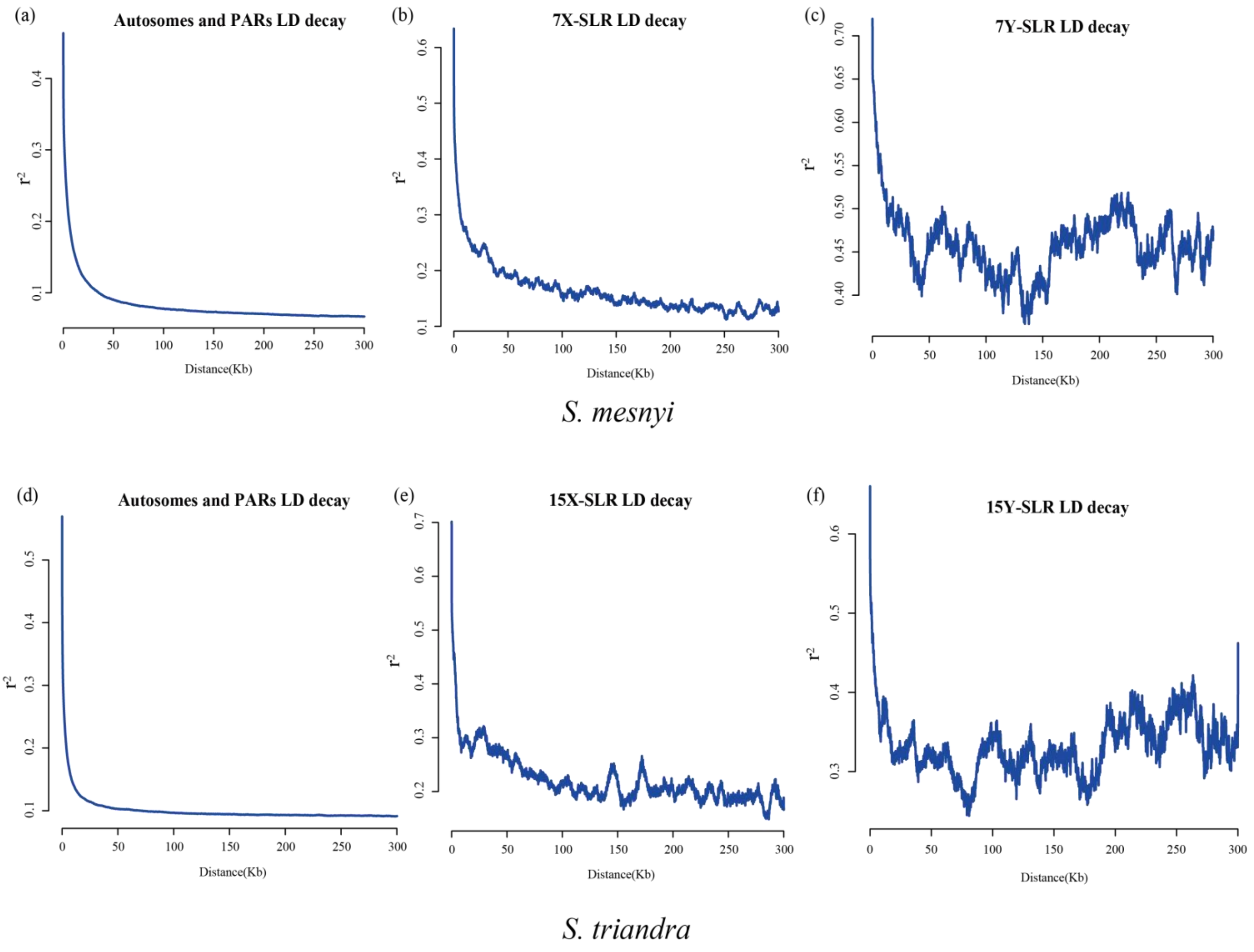

Figure S11

Collinearity between sex chromosomes and homologous autosomes in *Salix mesnyi*, *S. dunnii*, *S. arbutifolia* and *S. polyclona*. The magenta lines represent the sex-linked regions for each species, dark green indicates inversions.

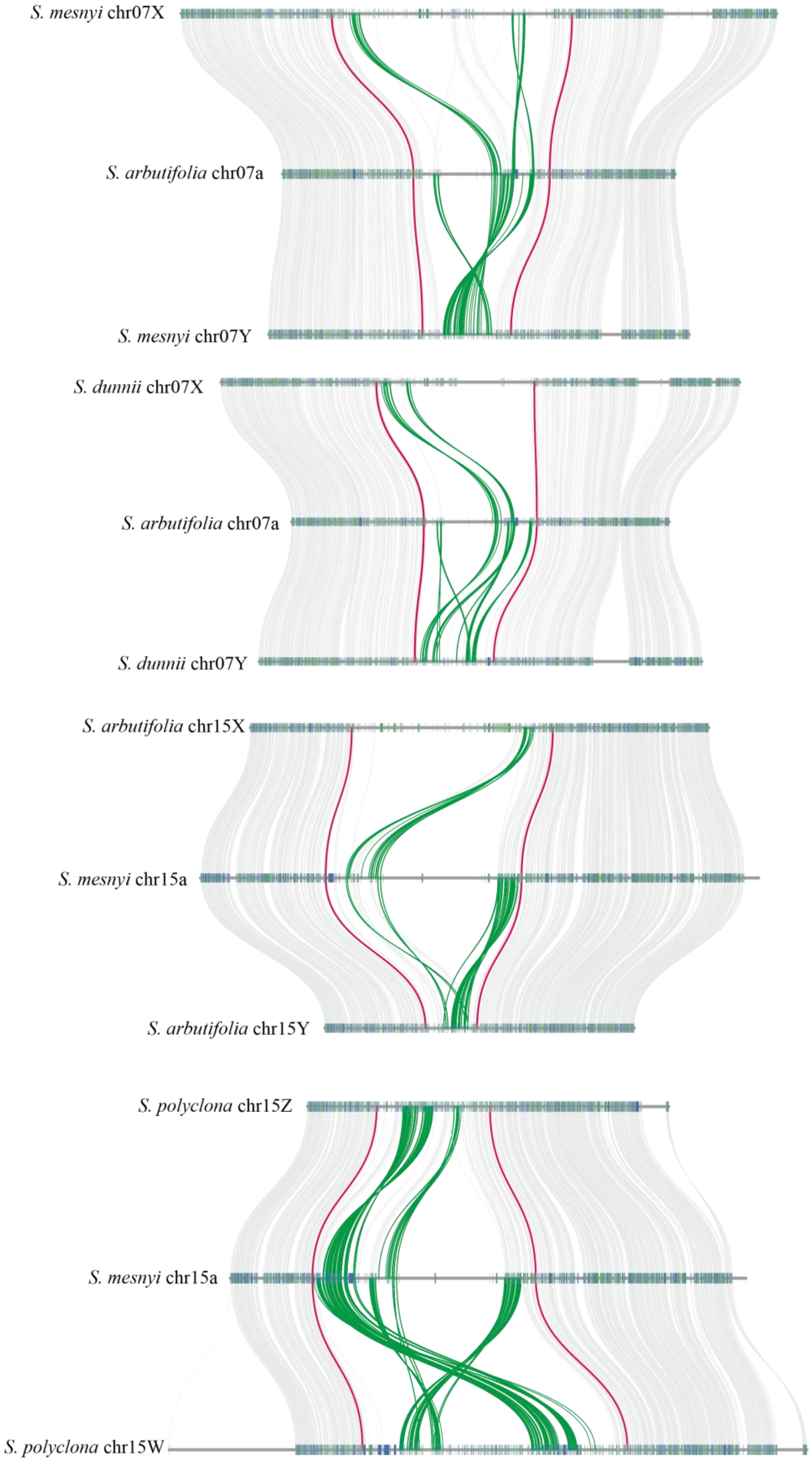

Figure S12

The collinearity analysis of homologous autosomes corresponding to the sex chromosomes in *Salix* (a) and *Vetrix* (b) clade species, respectively. Dark green indicates inversions.

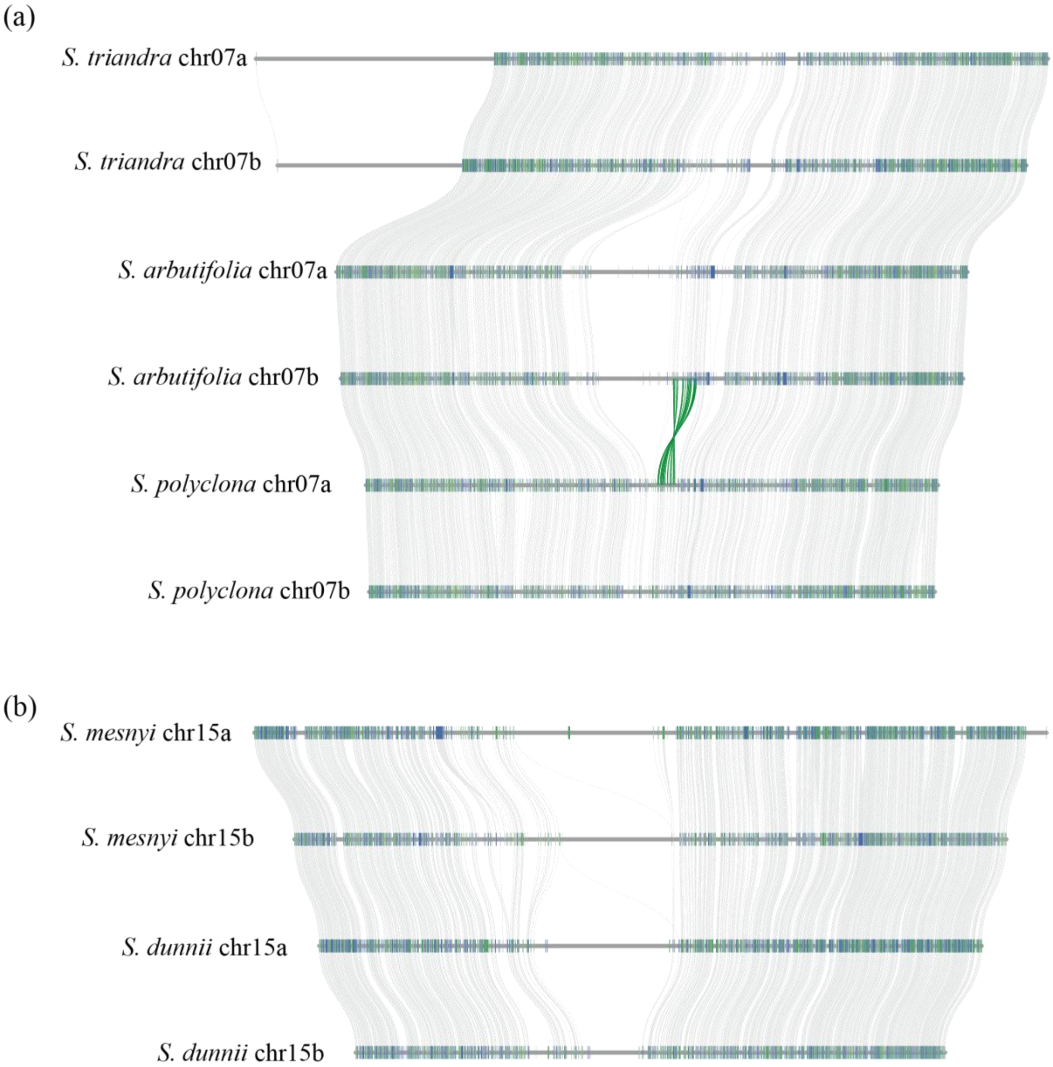

Figure S13

Inferred pericentromeric regions of *Salix mesnyi* autosome 15*a*, *S. triandra* autosome 7*a*, and *S. polyclona* autosomes 7*a* and 7*b*. They all contain gene density results, the total TE density, LTR-Gypsy density, LTR-copia density values. LD patterns (measured as r^2) were only applied to the newly sequenced *S. mesnyi* autosome 15*a* and *S. triandra* autosome 7*a* in this study. Light blue areas represent inferred pericentromeric regions. The green block in *S. polyclona* 7*a* and 7*b* represents the position of the inversion in Fig. S12a, which is located in the inferred pericentromeric region.

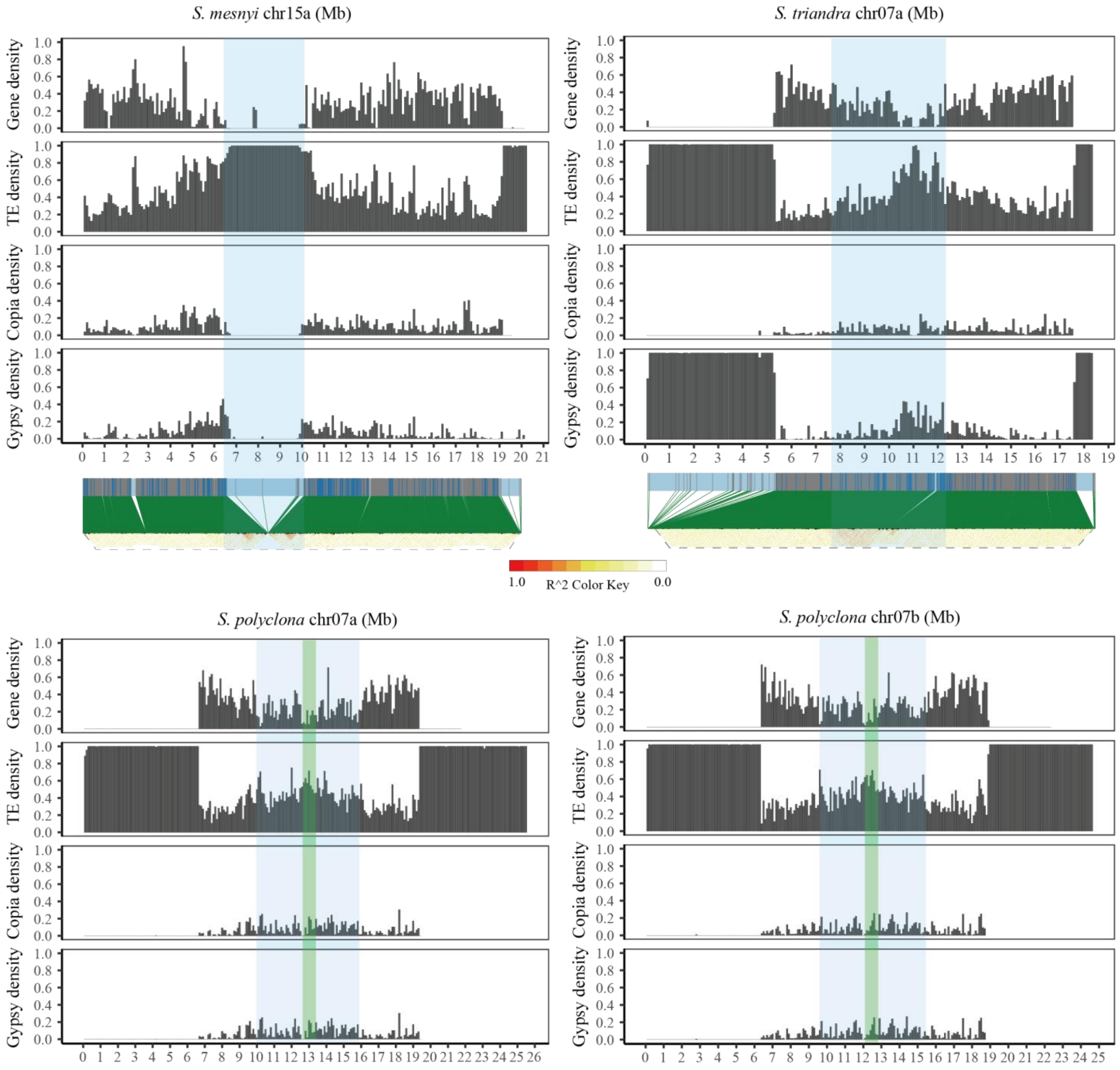

Figure S14

X expansion in two clades of *Salix* species. (a) shows the size of the X and Y-SLRs in different species; (b) illustrates the main elements of the X-extended region.

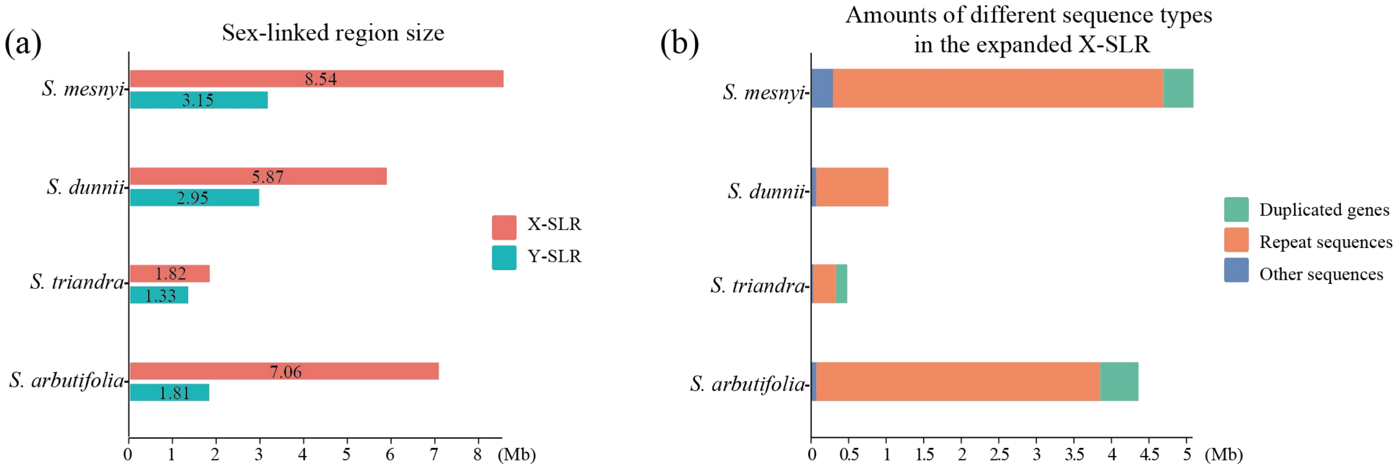

Figure S15

Percentages of ancestral SLR gene losses in the 7XY and 15XY systems.

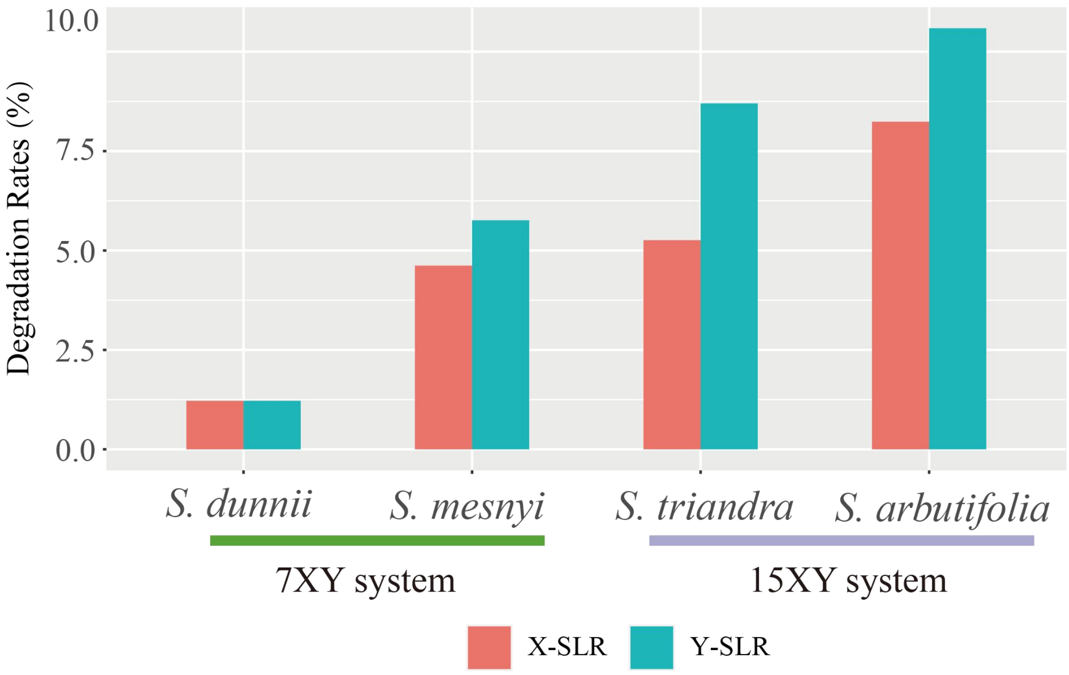

Figure S16

Expression patterns of exon 1 of *ARR17*-like duplicates and *MSF* genes of *Salix triandra*. (a-b) Transcription level of the two intact *ARR17*-like genes in male and female buds of chromosome 19*a* (a) and chromosome 19*b* (b) of *S. triandra*. (c) sRNA counts near exon 1 of *ARR17*-like partial duplicates in 15Y-SLR of *S. triandra*. P1-P7 represent *ARR17*-like partial duplicates 1-7 (exon 1). (d-e) Transcription levels of *MSF* and *PI*-like genes in buds and catkins of *S. triandra*.

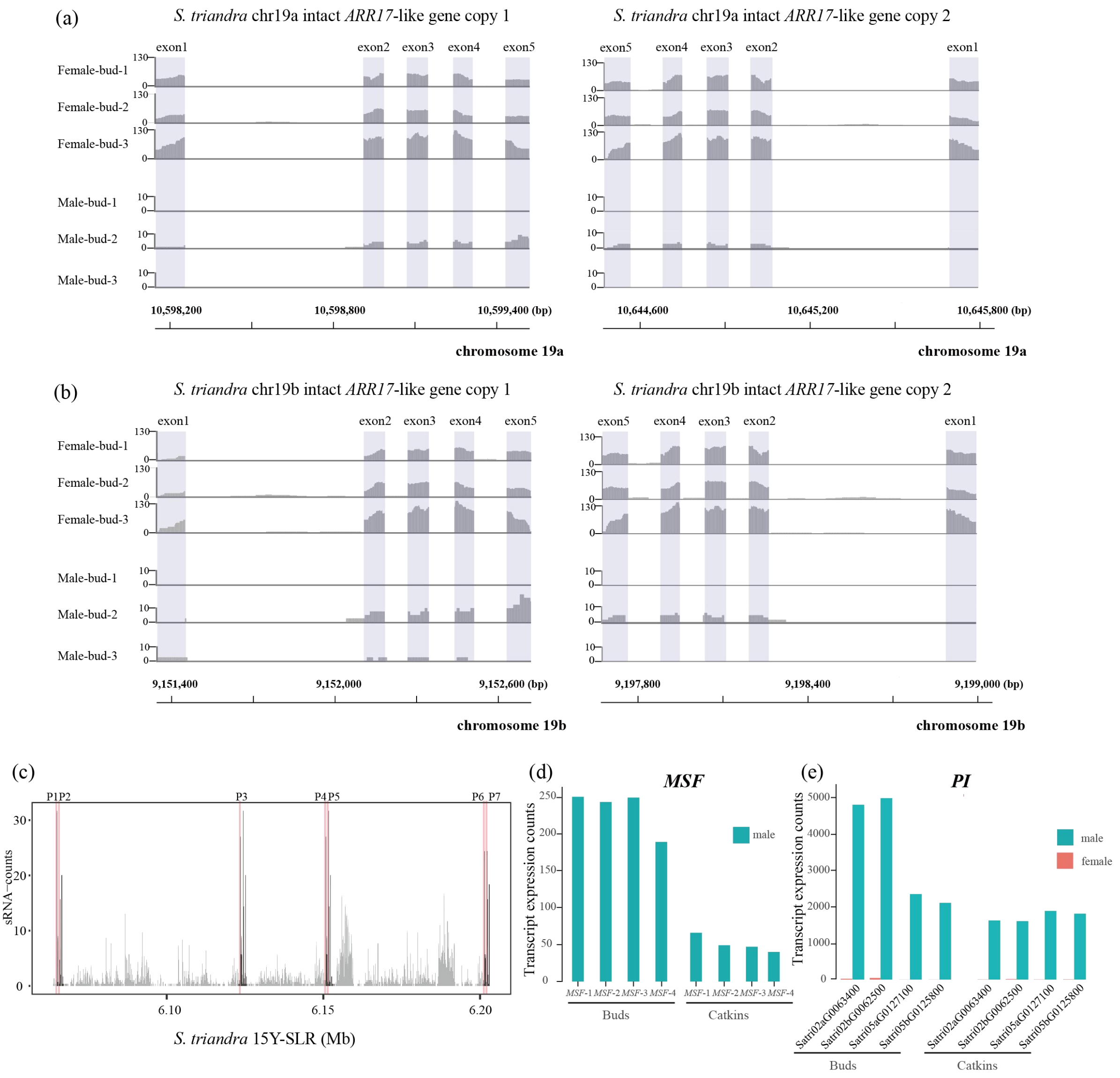

Supplement: msae235_Supplementary_Data [file msae235_supplementary_data.zip › Supplementary figures.pdf]
